# Construction of Triboelectric Series and Chirality Detection of Amino Acids Using Triboelectric Nanogenerator

**DOI:** 10.1002/advs.202307266

**Published:** 2023-11-30

**Authors:** Arnab Pal, Anindita Ganguly, Po‐Han Wei, Snigdha Roy Barman, Chia‐Chih Chang, Zong‐Hong Lin

**Affiliations:** ^1^ International Intercollegiate PhD Program National Tsing Hua University Hsinchu 30013 Taiwan; ^2^ Institute of Biomedical Engineering National Tsing Hua University Hsinchu 30013 Taiwan; ^3^ Department of Biomedical Engineering National Taiwan University Taipei 10617 Taiwan; ^4^ Department of Applied Chemistry National Yang Ming Chiao Tung University 1001 University Road Hsinchu 30010 Taiwan

**Keywords:** amino acids, chirality detection, contact electrification, racemization, triboelectric series, work function

## Abstract

Triboelectrification necessitates a frictional interaction between two materials, and their contact electrification is characteristically based on the polarity variance in the triboelectric series. Utilizing this fundamental advantage of the triboelectric phenomenon, different materials can be identified according to their contact electrification capability. Herein, an in‐depth analysis of the amino acids present in the stratum corneum of human skin is performed and these are quantified regarding triboelectric polarization. The principal focus of this study lies in analyzing and identifying the amino acids present in copious amounts in the stratum corneum to explain their positive behavior during the contact electrification process. Thus, an augmented triboelectric series of amino acids with quantified triboelectric charging polarity by scrutinizing the transfer charge, work function, and atomic percentage is presented. Furthermore, the chirality of aspartic acid as it is most susceptible to racemization with clear consequences on the human skin is detected. The study is expected to accelerate research exploiting triboelectrification and provide valuable information on the surface properties and biological activities of these important biomolecules.

## Graphical abstract

Triboelectrification relies on friction between two materials, exploiting the polarity variations in the triboelectric series for contact electrification. The study delves into amino acids in human skin's stratum corneum, quantifying their triboelectric properties. It emphasizes high‐abundance amino acids in the skin's outer layer, presenting an extended triboelectric series. Additionally, it identifies homochiral aspartic acid susceptible to the racemization process.

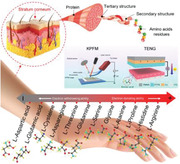

## 1 Introduction

Triboelectrification is well known for its universality and contact‐induced electrification.^[^ ^1^, ^2^, ^3^ ^]^ Depending on the circumstances, the consequences of this phenomenon can be very beneficial or irksome.^[^ ^4^, ^5^ ^]^ Due to the importance of contact electrification, it has been extensively exploited in various types of applications, such as green energy harvesting,^[^ ^6^, ^7^ ^]^ chemical sensing,^[^ ^8^, ^9^ ^]^ flexible electronics,^[^ ^10^, ^11^ ^]^ health care,^[^ ^12^, ^13^, ^14^ ^]^ air ion generation for the removal of air pollutants,^[^ ^15^ ^]^ artificial intelligence,^[^ ^16^ ^]^ and the Internet of Things (IoT)^[^ ^17^ ^]^ during the last decade. Thus, there is a strong motivation for an in‐depth study of diverse triboelectric materials. Amino acids, peptides, proteins, skins, wood, hair, and cotton are biomaterials that are characterized as triboelectric materials.^[^ ^1^, ^18^, ^19^, ^20^ ^]^ However, their electrification strengths are different, depending on their relative position in the triboelectric series. In this series, diverse materials are ranked according to their propensity to lose or gain electrons, reflecting the native physical properties of the materials.^[^ ^21^, ^22^ ^]^

Moreover, contact‐induced electrostatic interactions and triboelectrification are crucial in regulating several natural processes and biological functions (e.g., immune cells engulf pathogens, bumblebees pollinate flowers, and viruses contaminate host cells).^[^ ^23^, ^24^ ^]^ Amino acids, the essential macromolecules for life, play a fundamental role in several of these biological and physical processes. These interactions are closely dependent on biological assemblies, thus even the mutation of a single amino acid can entirely transform biological characteristics and functions leading to anomalies.^[^ ^25^, ^26^ ^]^ Human skin, besides other natural biomaterials, is also considered a prominent triboelectric material where the basic building blocks are the amino acids. It is believed to have a highly positive contact‐induced triboelectric property.^[^ ^1^, ^27^ ^]^ However, the availability of less comprehensive models to study their electrical properties at the molecular level owing to their complex nature, results in an elusiveness regarding the electrical properties of these biological materials.^[^ ^22^ ^]^ Moreover, in the case of amino acids the l‐forms have greater biological abundance than the d‐form.^[^ ^28^, ^29^ ^]^ Some of the rare d‐amino acids are biomarkers for infectious diseases, kidney diseases, cognitive disorders, and neurodegenerative diseases. Hence, the sensing of chiral molecules gains attention worldwide. However, existing systems such as circular dichroism (CD), optical rotatory dispersion (ORD), nanomaterial enhanced chirality sensing, etc.^[^ ^28^ ^]^ are restricted from extensive real environment use due to the requirement of trained operators, external power sources, and bulk size.

**Figure 1:**
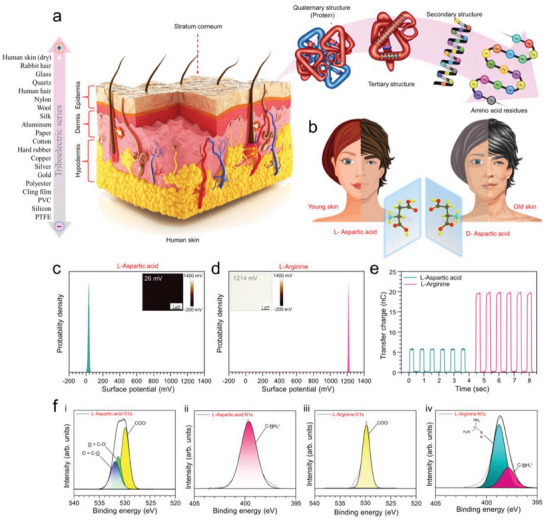
Characterization and triboelectric quantification of human skin constituent amino acids and their effect on aging skin. a) Schematic representation of human skin and human skin constituents. b) Effects of racemization of homochiral amino acids on the aging of human skin. c) Surface potential mapping and the average surface potential distribution curve of l‐aspartic acid by KPFM. d) Surface potential mapping and the average surface potential distribution curve of l‐arginine by KPFM. e) Measured transfer charge of l‐aspartic acid and l‐arginine against PTFE shows a distinct difference. f) Core level photoemission spectrum analysis of the O1s and N1s peaks of l‐aspartic acid and l‐arginine.

Herein, a breakthrough comes from a standardizing scheme that quantitatively measures the triboelectric charge density (TECD) of test materials in contact with a standard material by utilizing the output of a triboelectric nanogenerator (TENG) under static conditions to quantify the triboelectric series.^[^ ^1^, ^21^ ^]^ Here, the triboelectric transfer charge is measured by using polytetrafluoroethylene (PTFE) as a standard material to quantify the amino acids in an augmented triboelectric series. Further, by comparing the positions of the amino acids in the triboelectric series, the positive behavior of human skin under triboelectrification is conclusively explained (**Figure** **1**). According to previous reports, the contact electrification phenomenon is intimately related to the work function and the electron‐donating or electron‐accepting capabilities of the concerned materials.^[^ ^30^, ^31^, ^32^ ^]^ Thus, the work functions of the amino acids are quantitatively standardized by standard experimental and computational methods.^[^ ^33^, ^34^ ^]^ Kelvin probe force microscopy (KPFM) is employed to map the surface potential, revealing different work function values of the amino acids. Moreover, density functional theory (DFT) is utilized to predict the work functions using the CAmbridge Serial Total Energy Package (CASTEP) module of Materials Studio (MS) software. In addition, X‐ray photoelectron spectroscopy (XPS) characterization‐based elemental analysis reveals the atomic percentages of the constituent elements, predicting the electron‐donating or electron‐accepting capabilities.

In addition, the triboelectric identification of the chiral amino acid present in stratum corneum (SC) is performed as the triboelectric behavior of any biomaterial can be influenced by the change even in a single amino acid. For human skin, the racemization phenomena of l‐aspartic acid play an imperative role during the aging process.^[^ ^35^, ^36^, ^37^ ^]^ This spontaneous conversion of the enantiomeric state of the aspartic acid has been used as a dating method to estimate the aging process in mammals.^[^ ^36^ ^]^ This method relies on the fact that proteins in living animals and plants are almost entirely composed of l‐amino acids. However, the accumulation of d‐aspartic acid in unturned‐over proteins over time, resulting in changes in the physicochemical properties of affected proteins contribute to the progressive changes in the aging process, and the effects are prominent on the SC.^[^ ^37^ ^]^ Therefore, our study utilizes the triboelectric phenomenon to scrutinize two major aspects: the positive behavior of the human skin during the contact electrification process, and the successful detection of the chiral amino acids, which is solely responsible for producing protein conformational changes due to racemization over time and contribute to the geological aging process of human skin. A better understanding of the triboelectric properties of the amino acids can provide insights into the fundamental processes and enable the design of new materials and technologies.

## 2 Results

### 2.1 Characterizations of the Amino Acids with Charged and Polar Uncharged Side Chains

**Figure 2:**
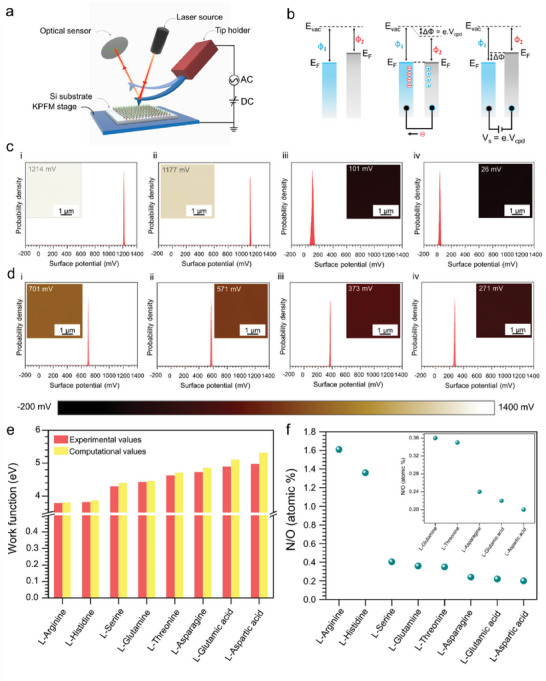
Characterization of the amino acids with charged and polar uncharged sidechains. a) Schematic illustration of the KPFM experiments. The amino acid samples are coated on a Si substrate and placed on the conductive KPFM stage. b) The working mechanism of KPFM. c) Surface potential mapping of amino acids with charged sidechains: i) l‐arginine, ii) l‐histidine, iii) l‐glutamic acid, and iv) l‐aspartic acid. d) Surface potential mapping of amino acids with uncharged polar sidechains: i) l‐serine, ii) l‐glutamine, iii) l‐threonine, and iv) l‐asparagine. e) Comparison of the experimental and computational work function values of the mentioned amino acids. f) N/O atomic percentages of the amino acids with charged and polar uncharged sidechains from the survey scan by XPS.

Amino acids can be classified based on the distinctive attributes of their sidechains. According to previous studies, the exclusive groups on the sidechain effectively determine the polarity of the material. The extent of electron transfer depends on the electron‐donating or electron‐accepting capabilities of each material, which vary depending on the specific sidechain, leading to contact electrification. The surface charge polarity is influenced by certain factors such as charge mosaics,^[^ ^22^, ^31^, ^38^, ^39^ ^]^ surface roughness,^[^ ^40^ ^]^ surface charge polarity,^[^ ^41^, ^42^ ^]^ and contact materials.^[^ ^43^, ^44^ ^]^ The concept of charge mosaics is yet to be experimentally proven. However, KPFM has been used to visualize these surface charge patterns, but it is yet to be ascertained whether this mosaic pattern applies universally to contact electrification involving various dielectrics and, if so, at what scales these mosaics manifest. The difference in the work function values between two materials plays a major role in contact electrification.^[^ ^22^ ^]^ Therefore, the transfer charge capabilities of these amino acids were inspected by measuring the work function. Herein, KPFM is utilized to perform surface potential mapping of the amino acids (**Figure** **2**a,b). The resultant surface potential mapping shows very distinct differences among the amino acids with charged sidechains, as depicted in Figure 2c. In addition, from Figure 2d the differences in surface potentials with work function values of the amino acids having polar uncharged sidechains are quite prominent. Moreover, work function values are predicted using DFT, validating the distinct crystal planes that appear in the X‐ray diffraction (XRD) analysis, as shown in Figures S1–S4 (Supporting Information). The predicted work function values are in good agreement with the obtained experimental values (Figure 2e).

To further investigate the relationships between the functional groups and the electron‐donating or electron‐accepting capabilities of the amino acids, XPS is utilized (Figures S5–S8, Supporting Information). From the XPS survey scan, the nitrogen‐based functional groups can be designated as electron‐donating groups; conversely, the oxygen‐based groups can be designated as electron‐accepting groups.^[^ ^45^, ^46^, ^47^ ^]^ Moreover, from Table S1 (Supporting Information), no significant change is observed in the atomic percentage of carbon in each molecule, while the main difference can be seen in the percentages of nitrogen and oxygen. Based on these data, our study is focused on the spectra obtained at N1s and O1s.

From the high‐resolution XPS analysis, the positive charge is obviously dislocated to the guanidine group of the sidechain in the zwitterionic form of l‐arginine, despite being at the α‐amine position. Thus, the N1s spectrum exhibits two peaks at 397.9 and 398.7 eV (Figure S5c, Supporting Information). The peak at 398.7 eV represents three nitrogen atoms, and the other peak represents the nitrogen of the amine group; however, l‐arginine has one peak in the O1s photoemission spectrum, confirming the existence of a carboxylate (COO─) group (Figure S5d, Supporting Information).^[^ ^48^, ^49^ ^]^ The sidechain of the l‐histidine comprises an imidazole group; thus, the high‐resolution N1s spectra contain three peaks. For C═N, the peak appears at 397.9 eV; for C–NH and protonated α‐amine nitrogen, the peaks are observed at 399.5 and 400.3 eV, respectively (Figure S5f, Supporting Information).^[^ ^48^ ^]^ Moreover, the O1s spectrum for l‐histidine is similar to that of l‐arginine (Figure S5g, Supporting Information). Furthermore, the spectra of l‐glutamic acid and l‐aspartic acid exhibit α‐nitrogen peaks at 399.5 and 399.6 eV, respectively. Nevertheless, both O1s spectra contain three peaks attributed to O═C─OH, O═C─OH, and COO^−^ (Figure S6c–h, Supporting Information).^[^ ^48^, ^49^ ^]^ Among the polar uncharged sidechain‐based amino acids, l‐serine and l‐threonine have hydroxyl groups, whereas the amide group is present in the sidechain of l‐asparagine and l‐glutamine. l‐Serine and l‐threonine have a single α‐amine nitrogen atom; thus, the photoelectron spectrum shows a solitary peak that belongs to C–NH_3_^+^, and there are two peaks in the O1s region, which can be attributed to the oxygen atoms in the carbonyl group (O═C─O) and hydroxyl group (OH─) (Figure S7c–h, Supporting Information).^[^ ^50^, ^51^ ^]^ Additionally, for l‐asparagine and l‐glutamine, the N1s spectra of these amino acids have two peaks, which are attributed to the nitrogen atoms in the NH_2_ and C─NH_3_^+^ groups; the O1s spectrum consists of two peaks representing the oxygen atoms in the O═C─O and OH─ groups (Figure S8c–h, Supporting Information).^[^ ^49^, ^52^ ^]^ Hence, through XPS analysis, the N/O ratios of different amino acids can be defined, justifying their electron‐donating ability. From Figure 2f, when the N/O ratio decreases, the work function of the corresponding amino acid increases.

### 2.2 Characterization of the Amino Acid with Special and Hydrophobic Sidechains

**Figure 3:**
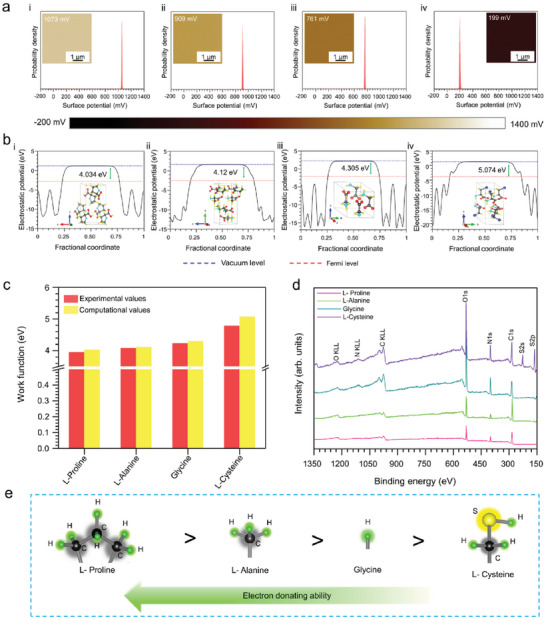
Characterization of the amino acids with special and hydrophobic sidechains. a) Surface potential mapping of i) l‐proline, ii) l‐alanine, iii) glycine, and iv) l‐cysteine. b) Estimated work functions by DFT calculation of i) l‐proline, ii) l‐alanine, iii) glycine, and iv) l‐cysteine. c) Comparison of the experimental and computational work function values of the amino acids. d) Survey spectra of l‐proline, l‐alanine, glycine, and l‐cysteine, where the N1s, O1s, C1s, S2p, and S2s peaks represent the different sidechain groups of the amino acids. e) Representation of the electron‐donating abilities of the sidechain groups of these amino acids.

Among the elementary amino acids, some have special kinds of sidechains that are different from others. The influences of these sidechain groups can be observed from the surface potential mapping (**Figure** **3**a) and computed work function values (Figure 3b) of these amino acids. The corresponding work functions are calculated from the average surface potential values (**Table** **1**). Furthermore, computational values (Figures S9,S10, Supporting Information) are compared in Figure 3C, which show excellent agreement. Moreover, the XPS survey scan (Figure 3d) and core‐level photoemission spectrum analysis confirm the presence of different functional groups in the amino acids (Figure S11,S12, Supporting Information), which can be arranged according to their electron‐donating ability (Figure 3e). Herein, glycine is the smallest achiral amino acid, and it has single nitrogen and oxygen atoms that induce single signals of the photoemission spectra of both atoms, confirming the presence of C–NH_3_^+^ and COO^−^ groups, respectively (Figure S11c,d, Supporting Information).^[^ ^48^, ^50^ ^]^ l‐Proline has an imino group belonging to the five‐member ring. Hence, the core level spectrum comprises two peaks, the dominant one representing the protonated secondary amino group (C–NH_3_^+^) and a neutral amino group (C─N) (Figure S11f,g, Supporting Information).^[^ ^53^ ^]^ In addition, l‐cysteine has a special thiol methyl/sulfhydryl sidechain group, which corresponds to the appearance of two additional peaks for S 2s and S 2p in the survey scan. The S 2p region comprises four peaks, where one pair of peaks, S 2p_3/2,_ is in the C─SH group (Figure S12c, Supporting Information).^[^ ^54^, ^55^, ^56^ ^]^ Nevertheless, l‐alanine is the simplest amino acid with enantiomers; from the photoemission spectrum, the presence of C–NH^3+^ and COO^−^ groups is confirmed (Figure S12g,h,, Supporting Information).^[^ ^57^ ^]^

**Table 1:** Measured surface potentials and the consecutive work function values of the amino acids.

Amino acid	Surface potential [mV]	Work function [eV]
l‐Arginine	1214	3.786
l‐Histidine	1177	3.823
l‐Proline	1056	3.944
l‐Alanine	909	4.091
Glycine	761	4.239
l‐Serine	701	4.299
l‐Glutamine	571	4.429
l‐Threonine	373	4.627
l‐Asparagine	271	4.729
l‐Cysteine	199	4.801
l‐Glutamic acid	101	4.899
l‐Aspartic acid	26	4.974

### 2.3 Quantification of the Triboelectric Series of Amino Acids and Validation of the Hypothesis

**Figure 4:**
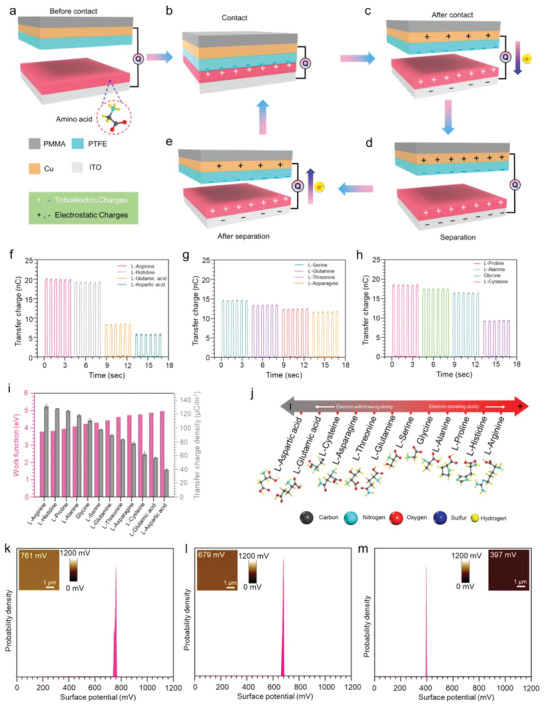
Quantification of the triboelectric series of amino acids based on their work function and transfer charge densities. Explanation and validation of human skin as a positive triboelectric material. a) Device structure of the solid–solid‐based TENG. b–e) Working principle of the solid–solid‐based TENG to investigate the charging behaviors of amino acids. f) Transfer charges of the amino acids with charged sidechain groups. g) Transfer charges of the amino acids with uncharged polar sidechain groups. h) Transfer charges of the amino acids with special and hydrophobic sidechain groups. i) Comparison of the work functions and transfer charge densities of the amino acids present in abundance in the SC. j) Augmented triboelectric series of amino acids with quantified triboelectric charging polarity by scrutinizing the triboelectric transfer charge, work function, and atomic percentage. k) Surface potential mapping and the average contact potential difference plot of silk fibroin, l) calf skin collagen, and m) gamma globulin.

To quantify the triboelectric series, the mechanisms of triboelectric nanogenerators (TENGs), have been utilized as the principle of measuring the triboelectric transfer charge based on contact electrification and electrostatic induction.^[^ ^58^, ^59^ ^]^ Furthermore, the triboelectric transfer charge density is calculated from the triboelectric transfer charge values. **Figure** **4**a depicts the fabricated basic structure of the TENG and the working process under short‐circuit conditions (Figure 4b–e) to measure the triboelectric transfer charge. At first, when two tribolayers contact each other, charge transfer occurs from the amino acid layer to the PTFE, as driven by the work function difference between them (Figure 4b). In the second stage, when they move from each other, to screen triboelectric changes, the electrons flow from the top electrode (PTFE side) to the bottom electrode (amino acid side) (Figure 4c). Next, when the gap distance is increased further to a limit (greater than ten times the thicknesses of the triboelectric layers), approximately all the electrons produced by contact electrification are transferred to the copper electrode, and charges from the copper electrode flow into the indium tin oxide (ITO) as triboelectric charges (Figure 4d). The PTFE is pressed toward the amino acid layer, and the number of induced charges on both electrodes will decrease, resulting in the backward flow of the electrons from the ITO electrode to the copper electrode (Figure 4e). Finally, when the amino acid layer and PTFE encounter each other, they return to the initial condition (Figure 4b). This conventional measurement method quantifies the TECDs of several categories of amino acids, where the obtained values imply variances in the basic charge transfer characteristics of the amino acids. Herein, Figure 4f–h depict the triboelectric transfer charges of amino acids with charged, polar‐uncharged, and special sidechains and a comparison of the characteristics, respectively. However, the analytical investigation remains an issue. For instance, the quantifiable amount of charge transferred is different for all the amino acids studied; some of the amino acids become highly positive, whereas others are less positively charged after contact and separation with the PTFE. We compare the respective TECD of different amino acids with their work function values (Figure 4i). Furthermore, we have compared the relative work functions of both contact materials that encounter contact electrification, as depicted in Figure S16 (Supporting Information). From Figure 4i, it is apparent that TECD is inversely proportional to the work functions of the amino acids. There is a simultaneous decrease in the value of the relative work function between the amino acids and the PTFE and TECD from 131.37 µC m^−2^ for l‐arginine to 39.59 µC m^−2^ for l‐aspartic acid (Figure S13, Supporting Information). In this work, as the reference material, PTFE is a highly negative triboelectric material with a very high work function (Φ_PTFE_ = 5.8 eV)^[^ ^60^ ^]^; thus, the polarity of the contact electrification charge is determined by the values of the relative work functions. As the TECD values are intimately related to the work function values, materials with greater differences in their work functions have more transferred electrons. Moreover, XPS analysis explains the reason for the variations in the work functions, which are related to the characteristics of the sidechain groups of these amino acids. Thus, an augmented triboelectric series of amino acids with quantified triboelectric charging polarity has been formulated by scrutinizing the triboelectric transfer charge, work function, and atomic percentage (Figure 4j) (The enlarged view of the triboelectric series is shown in Figure S14, Supporting Information). In this series, the amino acids present in copious amounts in the stratum corneum of the human skin (e.g., l‐serine, glycine, l‐alanine, l‐histidine, l‐threonine, and l‐proline) belong to a comparatively more positive side of the series than the other amino acids (Figure S15, Supporting Information). Hence, we imply that the cumulative effects of these amino acids make skin positive when it encounters contact electrification with other materials. For further validation of the hypothesis, we analyze some proteins, such as silk fibroin, calf skin collagen, and gamma globulin, under KPFM (Figure 4k–m). From the results, when the numbers of relatively positive amino acids (Figure S16, Supporting Information) are greater, the work function of the protein is less. Hence, this meticulous study validates the concept that the position of constituent amino acids defines the polarity of human skin (stratum corneum) or proteins after contact electrification.

### 2.4 Detection of Homochiral Amino Acids

**Figure 5:**
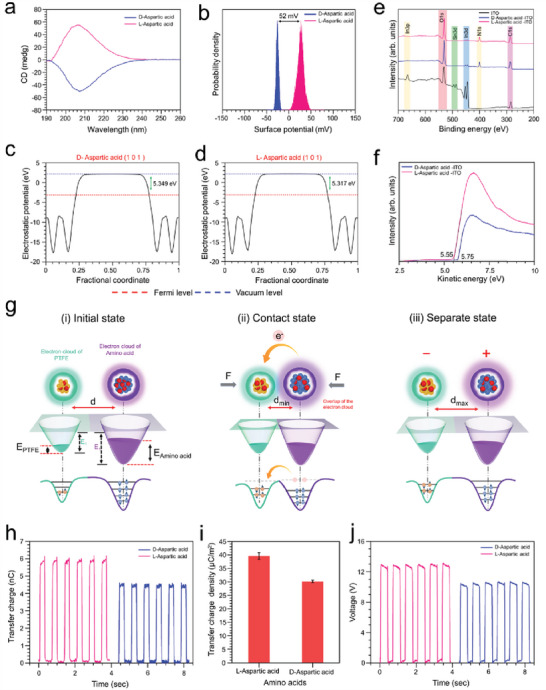
Detection of the homochiral amino acids takes part in the human skin aging process. a) The CD spectra analysis of the aqueous solution of the amino acids confirms the chirality of the used amino acid samples. b) The contact potential difference of the l‐ and d‐aspartic acid shows a distinct difference between their work function values. c,d) The simulated work function values of the l‐ and d‐aspartic acid by using DFT calculations. e) XPS survey scan of amino acids coated on the ITO glass to prepare the TENG layer, which confirms the successful coating. f) The measured Ultraviolet photoelectron spectroscopy (UPS) spectra with photon energy 21.22 eV on the ITO electrode coated with l‐ and d‐aspartic acid. g) Schematic representation of the electron‐cloud/potential‐well model of the contact electrification process. h,i) The measured transfer charge and transfer charge density in the short circuit condition. j) The measured voltage output in the open circuit condition.

Homochirality is indispensable for human life. During the primitive period of the origination of life on earth, for protein formation, only l‐amino acids are considered. Nevertheless, d‐amino acids, the optical enantiomers of l‐amino acids, have recently been discovered in various living systems. Residues of d‐aspartic acid have been detected in various extracellular proteins from diverse tissues in elderly individuals. Notably, d‐amino acid‐containing proteins are derived from tissues that are metabolically inert. Thus, throughout the course of an individual's life, d‐amino acid residues appear due to the racemization of l‐amino acids. However, aspartic acid is the most sensitive to racemization among all naturally occurring amino acids, and it is responsible for skin aging. Moreover, the amount of d‐aspartic acid increases with aging and causes elastosis, sagging, and wrinkling of the skin. Hence, sensing and detection of l‐and d‐aspartic acids are very important. Herein, a solid‒solid contact electrification‐based self‐powered technique has been exploited to detect l‐ and d‐aspartic acid, as depicted in **Figure** **5**.

The circular dichroism analysis of the aqueous solution of the amino acids confirms the chirality of the used amino acid samples (Figure 5a). The surface potential mapping of the L and D forms of aspartic acid via KPFM reveals distinct differences between the average surface potential values and the work functions of l‐ and d‐aspartic acid (Figure 5b). Moreover, the predicted work function values by the computational method of these enantiomers further ratify the difference (Figure 5c–d). According to the charge transfer mechanism of the TENG, molecules with different work function values provide different electrical outputs during contact electrification. Based on this concept, TENG devices are fabricated using l‐ and d‐aspartic acid. The devised TENGs have the same basic structure as the materials in Figure 4a, where the enantiomers are coated on the ITO surface. The XPS survey scan depicted in Figure 5e confirms the successful coating of amino acids on the ITO layers. Furthermore, the electronic properties of l‐ and d‐aspartic acid are characterized by work function measurements using ultraviolet photoelectron spectroscopy (UPS) after being coated on ITO (Figure 5f). The experimental values further confirm that the d‐aspartic acid layer possesses a higher work function value than the l‐aspartic acid layer. After being influenced by the differences in the work functions, different amounts of charge transfer can distinctly identify different molecules during contact electrification, which can be explained by the potential well/electron cloud model as depicted in Figure 5g. The difference in the triboelectric transfer charge and TECD values for l‐ and d‐aspartic acid leads to the efficient detection of these chiral amino acids (Figure 5h–i). Moreover, the measured voltage output confirms the detection of the enantiomers in the open circuit condition (Figure 5j).

## 3 Conclusion and Outlook

The surface potential mapping by KPFM reveals the distinct differences in the work functions of amino acids. By utilizing XPS characterization, we explain the reason behind the work function differences among the amino acids via the atomic percentage and presence of various sidechain functional groups. Moreover, the triboelectric transfer charge measurement paves the way for quantifying the augmented triboelectric series of the amino acids in the stratum corneum at a copious amount. Through the quantified triboelectric series, we have explained why human skin becomes positive during contact electrification. Finally, based on the contact electrification mechanism, our fabricated TENG device enables the detection of chiral amino acids undergoing racemization during the lifetime of humans. Thus, our study is highly expected to pave the way for the triboelectric quantification and detection of numerous biomaterials, which further expands the horizon of this field.

## 4 Experimental Section

### Chemicals

l‐Arginine (C_6_H_14_N_4_O, ≥ 98%), l‐glutamic acid (C_5_H_9_NO_4_, ≥ 99%), l‐proline, (C_5_H_9_NO_2_, 99%), l‐asparagine (C_4_H_8_N_2_O_3_, 99%), l‐glutamine (C_5_H_10_N_2_O_2_, ≥ 98%), l‐aspartic acid (C_4_H_7_NO_4_, ≥98%), and l‐threonine (C_4_H_9_NO_3_, ≥ 98%) were bought from Sigma–Aldrich; l‐histidine (C_6_H_9_N_3_O_2_, 99%), and l‐serine (C_3_H_7_NO_3_, 99%) were purchased from Alfa Aesar. l‐Alanine (C_3_H_7_NO_2_, 99%), l‐cysteine (C_3_H_7_NO_2_S, 99%), and glycine (C_2_H_5_NO_2_, 99%) were purchased from Acros Organics. d‐aspartic acid (99%) was purchased from Sigma–Aldrich. Silk fibroin was purchased from ChemSrc, collagen (calf skin) was purchased from Merck, and gamma globulin (bovine blood) was purchased from Sigma–Aldrich. Deionized (DI) water from a Milli‐Q ultrapure system and acetone from Echo Chemical Co. were used throughout the experiments. By using high‐purity deionized water (18.2 MΩ cm^−1^), all aqueous solutions were prepared. Without any further modification, all the chemicals were used as received. The single‐side polished highly doped p‐type (1 0 0) Silicon wafer was purchased from Twice Jin Limited, Taiwan. The thickness of the Si wafer is 675 ± 25 µm and the resistivity is 1–10 ohm‐cm.

### Fabrication of the TENG Device

To engineer the TENG, first, pieces of ITO glass slides (1 cm × 1.5 cm × 0.04 cm) were ultrasonically cleaned by immersion in DI water, acetone, and ethanol for 10 min each. Then, the glass slides were dried by using a nitrogen gas jet. Subsequently, the conductive surfaces of the ITO glasses were treated with oxygen plasma at 28 W power with 6.7 × 10^−1^ Bar O_2_ gas pressure for 5 min (Harrick Plasma, USA, 28 W). In the next step, the aqueous amino acid solutions of 25 mm were coated on the hydrophilic ITO surface by the spin coating method at 200 rpm for 30 sec and 1000 rpm for 10 sec, making the ≈400 nm thick uniform film. After the coating, the samples were dried for 5 h at 37 °C. To construct the counter electrode with a standard material layer, the copper foil was attached to a poly (methyl methacrylate) (PMMA) substrate; then, a PTFE film (1 cm × 1.5 cm × 0.013 cm) was adhered onto the copper electrode. Copper wires were used for the electrical connection.

### Characterization

The amino acid crystallinity was analyzed using a powder X‐ray diffractometer (XRD; Rigaku TTRAX Ш). For chemical characterization and atomic percentage measurement, X‐ray photoelectron spectroscopy (XPS; ESCA Laboratory 250Xi, Thermo Fisher Scientific) was conducted with a PHI VersaProbe II. CASA XPS software was used for the deconvolution of the high‐resolution core‐level spectra. Furthermore, UPS of the chiral amino acids was measured by an ESCALAB 250 Xi spectrometer using HeI resonance lines (21.2 eV) under ultrahigh vacuum conditions.

### KPFM

Amplitude‐modulated Kelvin probe force microscopy (AM‐KPFM; BRUKER ICON with ScanAsyst) was utilized to probe the amino acid surface potential mapping. In this regard, we utilized a single crystal diamond‐based conductive atomic force microscopy (AFM) tip (AD‐2.8‐AS) (supplied by Adama Innovations). The tip was calibrated by using the highly oriented pyrolytic graphite (HOPG) standard sample (BRUKER). All KPFM measurements were performed under ambient conditions.

### Work Function Calculation

The determination of the work function (WF) in this study was accomplished via the measurement of contact potential difference (CPD) between a cantilever probe, single crystal diamond‐based conductive AFM tip (AD‐2.8‐AS), and the various samples of interest under investigation. The WF values of both the AD‐2.8‐AS tip and the sample were calculated employing Equation (1):

(1) $$\mathrm{CPD}=\left(\Phi_{\mathrm{tip}}-\Phi_{\mathrm{sample}}\right)/e$$

Herein, *Φ*_tip_ represents the work function of the AD‐2.8‐AS, *Φ*_sample_ signifies the work function of the sample's surface, and “e” denotes the elementary charge of an electron. To ensure accurate calibration, the WF of the single crystal diamond‐based conductive AFM tip (AD‐2.8‐AS) tip was calibrated against a highly oriented pyrolytic graphite (HOPG) reference sample with a known work function (*Φ*_HOPG_ = 4.6 eV).^[^ ^61^, ^62^, ^63^ ^]^ Consequently, the work function of the tip (*Φ*_tip_) was determined to be 5.00 eV.

The work function of the sample's surface (*Φ*_sample_) can be computed using Equation (2):

(2) $$\Phi_{\mathrm{sample}}=\Phi_{\mathrm{tip}}-e\times {\mathrm{CPD}}_{\mathrm{sample}}$$

In this equation, *Φ*_sample_ is the work function of the sample, *Φ*_tip_ represents the work function of the AD‐2.8‐AS tip, and *CPD*_sample_ corresponds to the contact potential difference measured for the sample under investigation. This methodology enables the accurate determination of the work function of the sample's surface, a critical parameter in understanding its electronic properties and surface potential. For each amino acid, at least three sets of samples were characterized and, on each sample, at least three positions were selected to confirm the repeatability of the measurements. Herein, all the amino acids are coated on the conductive side of the highly doped p^+^‐Si (1 0 0) substrate and the conductive side is grounded.^[^ ^64^, ^65^ ^]^

### The Setup of Electrical Characterization

The solid‒solid TENG was operated under a double‐electrode configuration in which the amino acid layer on the ITO was one contact surface and the PTFE film on the copper foil was the surface. The triboelectric transfer charge was measured by using an electrometer (Keithley Model 6514). A programmable linear motor was employed to perform the contact separation motion. Herein, the linear motor is operated with a force is 1 N, at a frequency of 2 Hz. The separation distance is 1 cm, the separation speed is 0.0256 m sec^−1^, and the mechanical waveform for the linear motor is a sin wave, which involves the back‐and‐forth movement of the TENG. Moreover, the contact area of the TENG is (1 × 1.5) cm^2^.

Furthermore, certain reports have shown that humidity and temperature can affect the triboelectric output and the surface potential measurements.^[^ ^66^, ^67^, ^68^, ^69^ ^]^ These studies have shown that a high relative humidity can affect the triboelectric output as the charge transfer in a triboelectric nanogenerator occurs through an electron pathway.^[^ ^67^, ^68^ ^]^ The relative humidity was maintained at a range of 30–50% for an optimized output. Similarly, based on these studies, we have performed the experiments in a controlled environment, where a dehumidifier was used to maintain the humidity at ≈40 ± 1% in case of the atomic force microscopy (AFM) and kelvin probe force microscopy (KPFM) measurements (Figure S17a, Supporting Information). Furthermore, for the electrical output measurements, the experimental setup was placed inside a polymethyl methacrylate (PMMA) enclosure with continuous nitrogen gas flow to maintain the humidity ≈40 ± 1%. The temperature was maintained at 25 ± 1 °C by utilizing the air conditioning system. We have attached the digital images of the experimental setup in Figure S17b (Supporting Information).

### Calculation Setup

For the computational prediction of the work functions of different amino acids, DFT calculations were implanted in the CASTEP module of Material Studio software. The generalized gradient approximation (GGA) with Perdew–Burke–Ernzerhof (PBE) was employed to describe the exchange‐correlation energy. Additionally, 381 eV was the cutoff energy of the plane‐wave basis. Moreover, with the on‐the‐fly generated (OTFG) ultrasoft (default) pseudopotentials, the coarse quality was used for the k‐points, and the Broyden–Fletcher–Goldfarb–Shannon (BFGS) algorithm was used for energy minimization.^[^ ^33^ ^]^ The convergence thresholds were 5 × 10^−5^ eV per atom for the total energy, and the interionic displacement was 0.005 Å. Moreover, a vacuum length of 20 Å along the *z*‐axis was used in all the crystals for the computation. All crystal structures were downloaded from the Cambridge Crystallographic Data Centre (CCDC via www.ccdc.cam.ac.uk/data_request/cif.).

## Conflict of Interest

The authors declare no conflict of interest.

## Author Contributions

Z.H.L. conceived the research idea and supervised the project. A.P. designed all the experiments, conducted the computational study, and fabricated the devices. A.P. and A.G. performed the material characterizations. P.H.W., C.‐C.C. conducted the circular dichroism experiments. S.R.B. and A.G. helped with the figure arrangement. Z.H.L. and A.P. analyzed the results and wrote the manuscript. All the authors discussed the results and commented on the manuscript.

## Supporting information

Supporting Information

## Data Availability

The data that support the findings of this study are available from the corresponding author upon reasonable request.
