# Supplementary material for: Construction of Triboelectric Series and Chirality Detection of Amino Acids Using Triboelectric Nanogenerator
Source: Adv Sci (Weinh). 2023 Nov 30;11(4):2307266. doi: 10.1002/advs.202307266 (PMC10811508; doi:10.1002/advs.202307266)
Supplement: Supplementary file 1 — Supporting Information [file ADVS-11-2307266-s001.pdf]

ADVANCED SCIENCE
Open Access

Supporting Information

for *Adv. Sci.*, DOI 10.1002/advs.202307266

Construction of Triboelectric Series and Chirality Detection of Amino Acids Using Triboelectric Nanogenerator

*Arnab Pal, Anindita Ganguly, Po-Han Wei, Snigdha Roy Barman, Chia-Chih Chang and Zong-Hong Lin**

Supporting Information

Amino acid	At. % of N	At. % of O	At. % of C
L-Arginine	25.49	15.82	58.82
L-Histidine	23.17	17.71	59.13
L-Serine	11.96	29.62	58.41
L-Glutamine	11.02	30.65	58.33
L-Threonine	10.21	28.98	60.81
L-Asparagine	07.66	31.59	60.75
L-Glutamic acid	07.44	34.42	58.14
L-Aspartic acid	08.58	41.74	49.69

Table S1. Atomic percentages of amino acids with charged and polar uncharged sidechains.

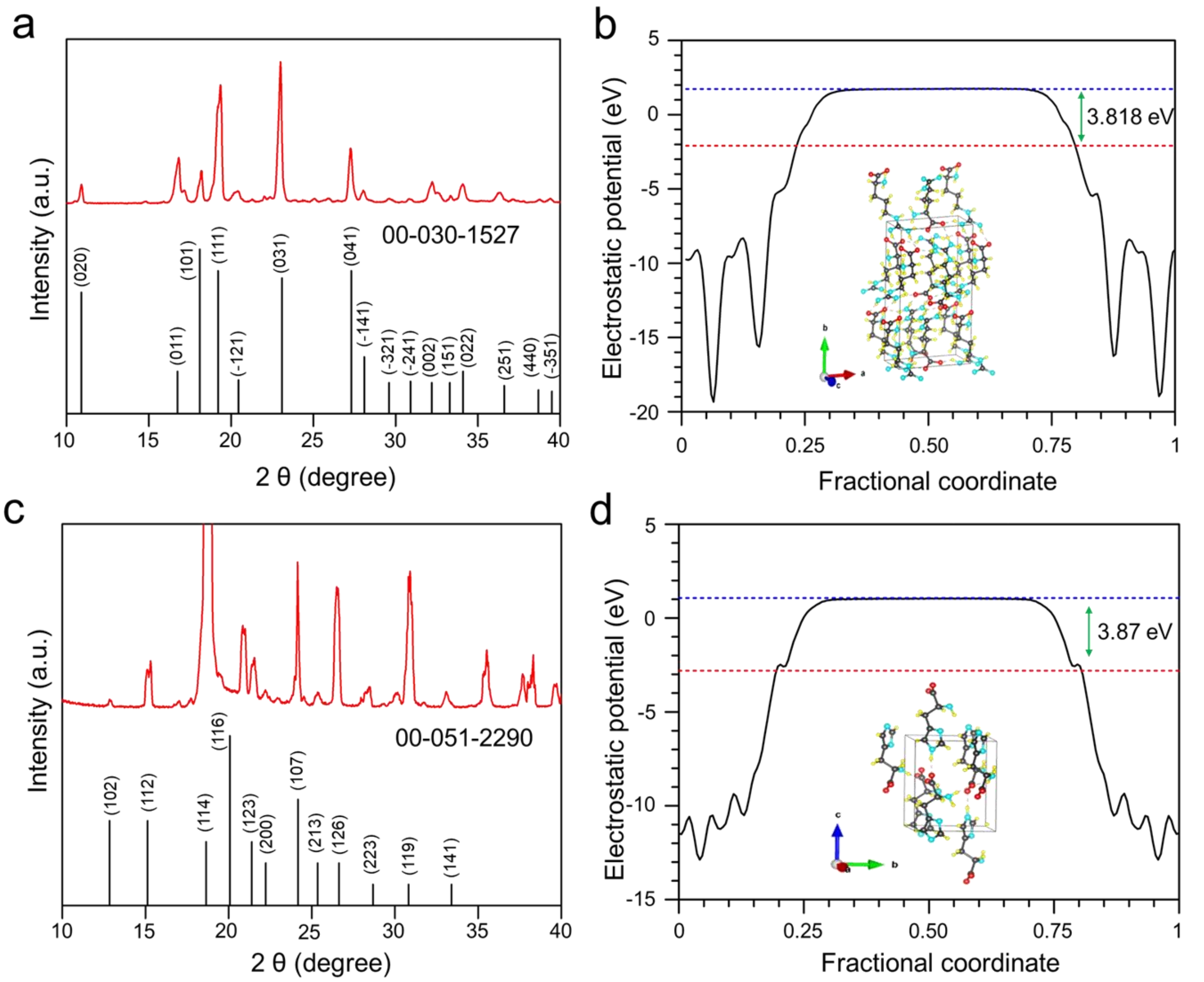

Figure S1. Characterization and work function estimation by the DFT calculations of amino acids with positively charged sidechains. XRD patterns of a) L-arginine and c) L-histidine. The simulated work function and unit-cell structure of b) L-arginine (0 3 1) and d) L-histidine (1 0 2).

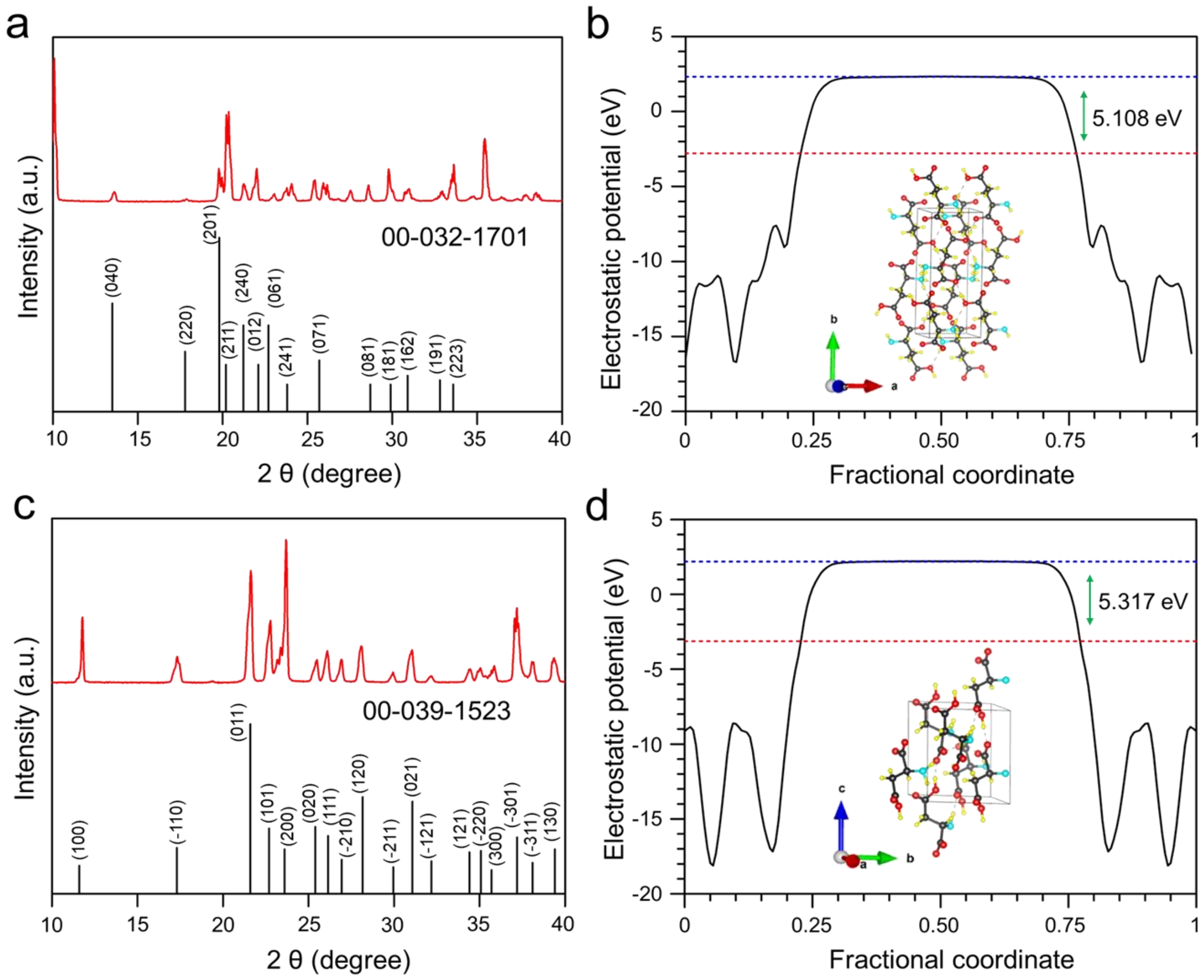

Figure S2. Characterization and work function estimation by the DFT calculations of amino acids with negatively charged sidechains. XRD patterns of a) L-glutamic acid and c) L-aspartic acid. The simulated work function and the unit-cell structure of b) L-Glutamic acid (0 1 2) and d) L-aspartic acid (1 0 1).

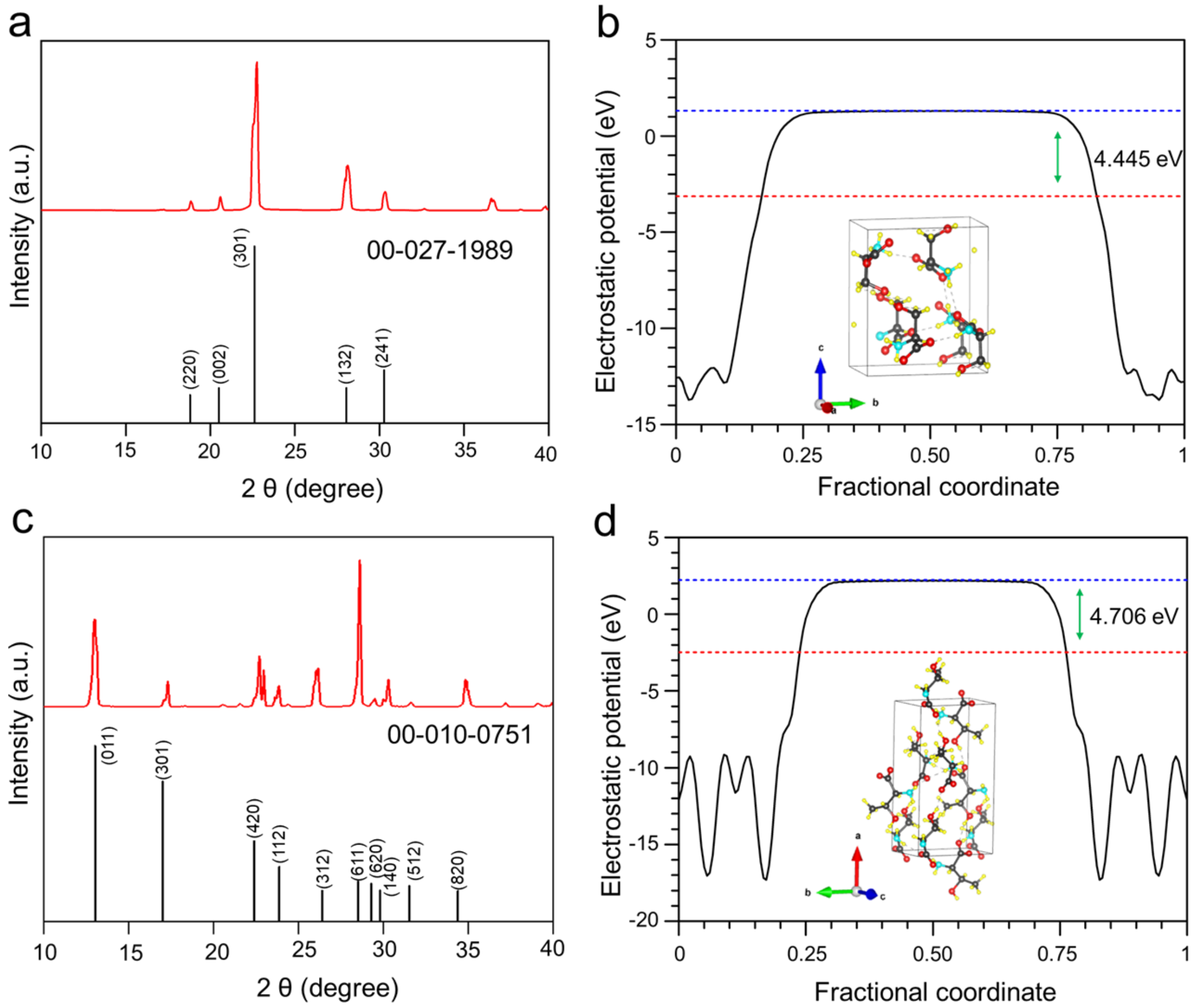

Figure S3. Characterization and work function estimation by the DFT calculations of uncharged polar amino acids. XRD patterns of a) L-serine and c) L-threonine. The simulated work function and the unit-cell structure of b) L-serine (1 3 2) and d) L-threonine (0 1 1).

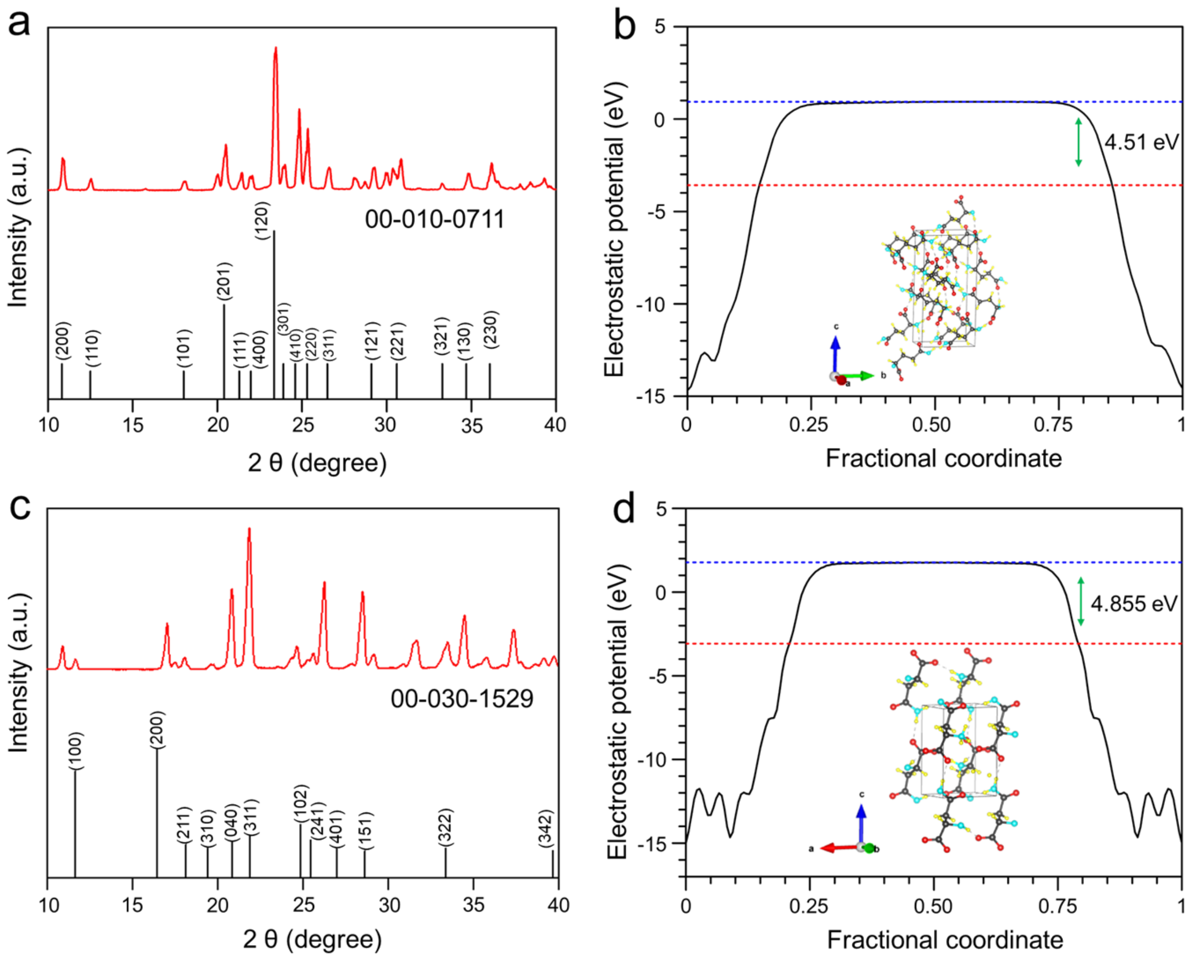

Figure S4. Characterization and work function estimation by the DFT calculations of uncharged polar amino acids (amide sidechain). XRD patterns of a) L-glutamine and c) L-asparagine. The simulated work function and the unit-cell structure of b) L-glutamine (3 1 1) and d) L-asparagine (1 0 2).

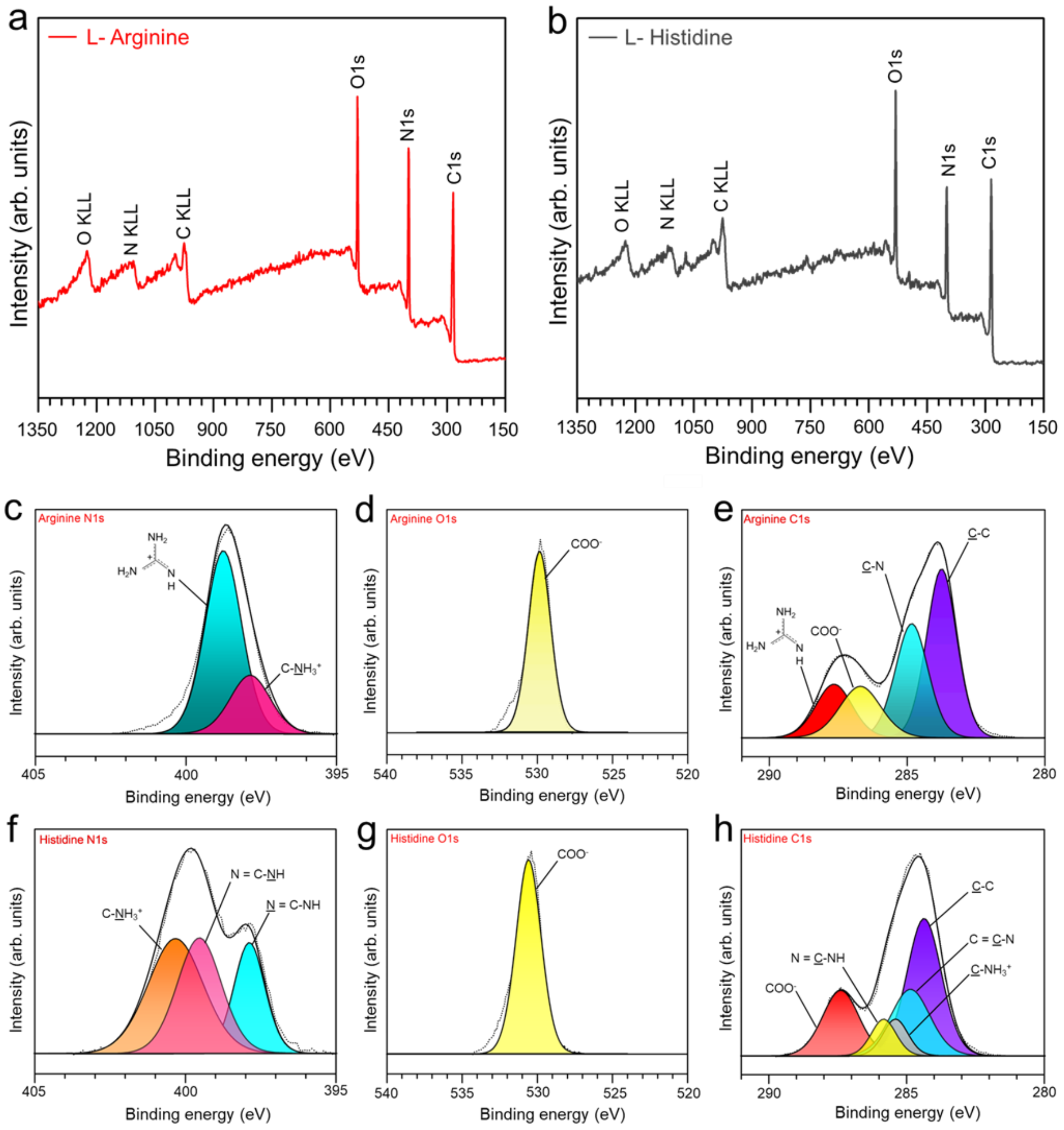

Figure S5. XPS characterization of amino acids with positively charged sidechains. Survey spectra of a) L-arginine and b) L-histidine. High-resolution core level photoemission spectra c) N1s of L-arginine, d) O1s of L-arginine, e) C1s of L-arginine, f) N1s of L-histidine, g) O1s of L-histidine, and h) C1s of L-histidine. Herein, black dots represent experimental data, whereas by summing the Gaussian–Lorentzian fits related to respective components, the final fit was obtained and represented by the continuous bold black line (various moieties are

represented in different colors under the spectra).

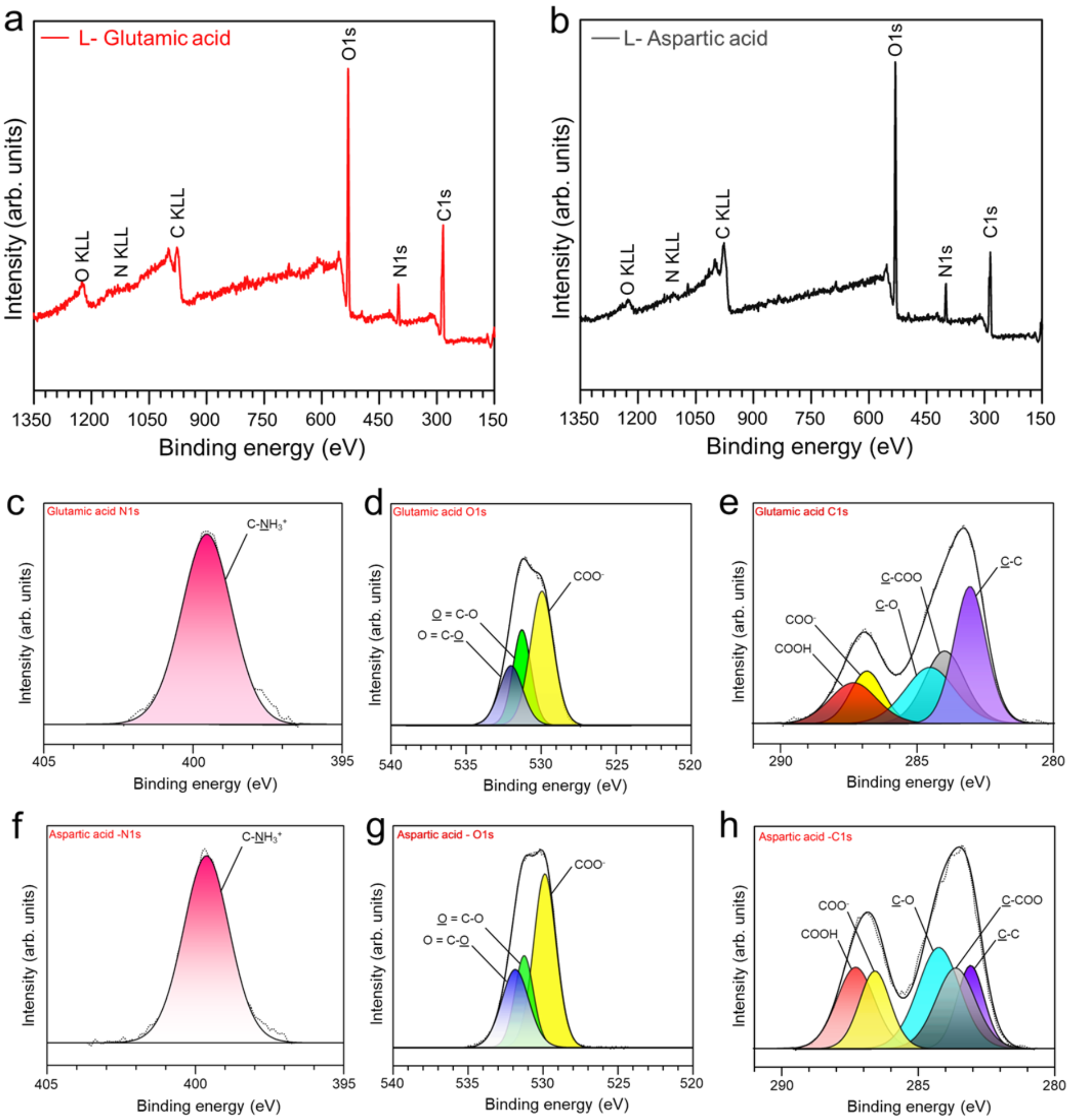

Figure S6. XPS characterization of amino acids with negatively charged sidechains. Survey spectra of a) L-glutamic acid and b) L-aspartic acid. High-resolution core level photoemission spectra c) N1s of L-glutamic acid, d) O1s of L-glutamic acid, e) C1s of L-glutamic acid, f) N1s of L-aspartic acid, g) O1s of L-aspartic acid, and h) C1s of L-aspartic acid. Herein, black dots represent experimental data, whereas by summing the Gaussian–Lorentzian fits related to respective components, the final fit was obtained and represented by the continuous bold black line (various moieties are represented in different colors under the spectra).

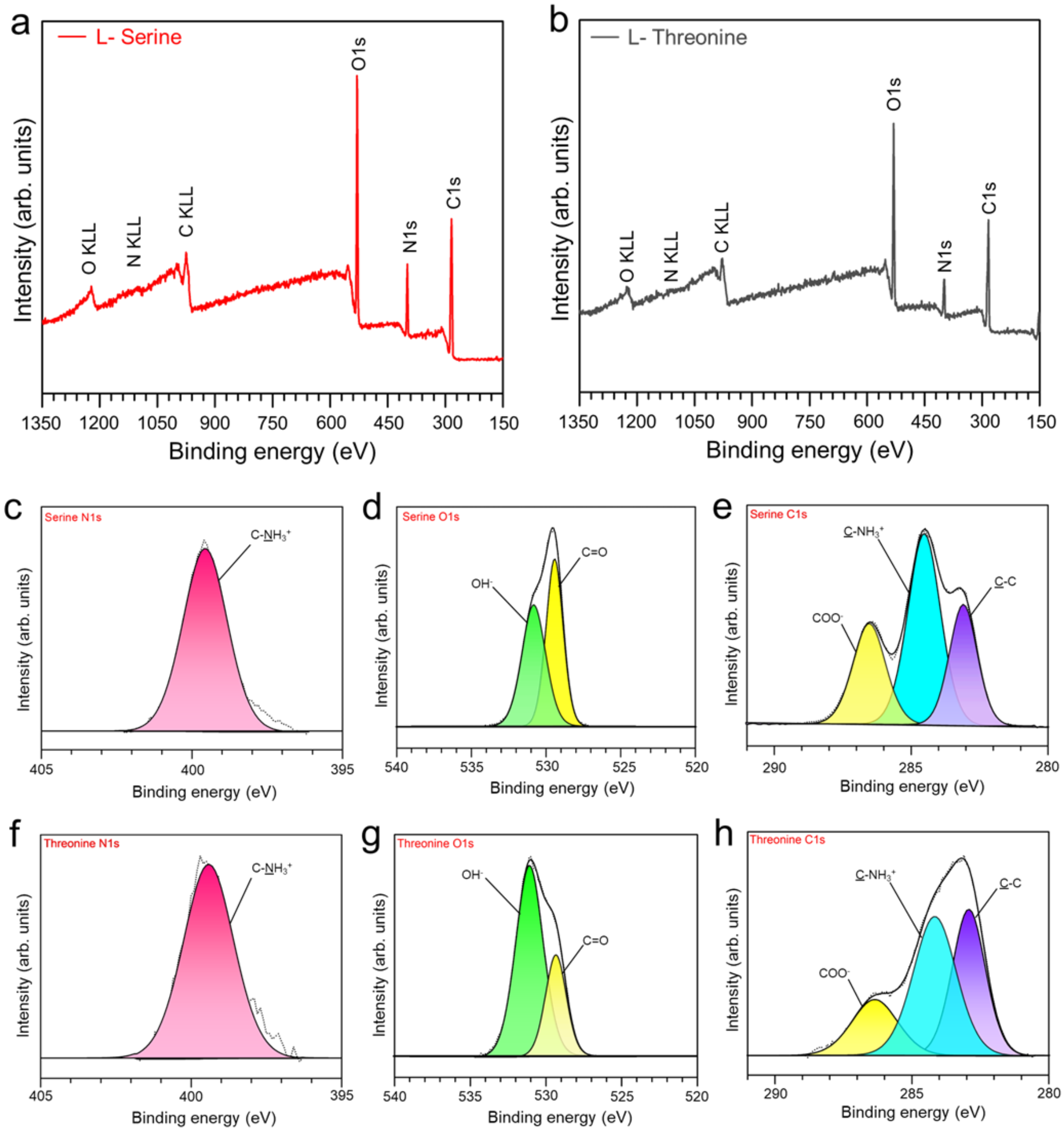

Figure S7. XPS characterization of amino acids with polar uncharged sidechains. Survey spectra of a) L-serine and b) L-threonine. High-resolution core level photoemission spectrum c) N1s of L-serine, d. O1s of L-serine, e) C1s of L-serine, f) N1s of L-threonine, g) O1s of L-threonine, and h) C1s of L-threonine. Herein, black dots represent experimental data, whereas by summing the Gaussian–Lorentzian fits related to respective components, the final fit was obtained and represented by the continuous bold black line (various moieties are represented in different colors under the spectra).

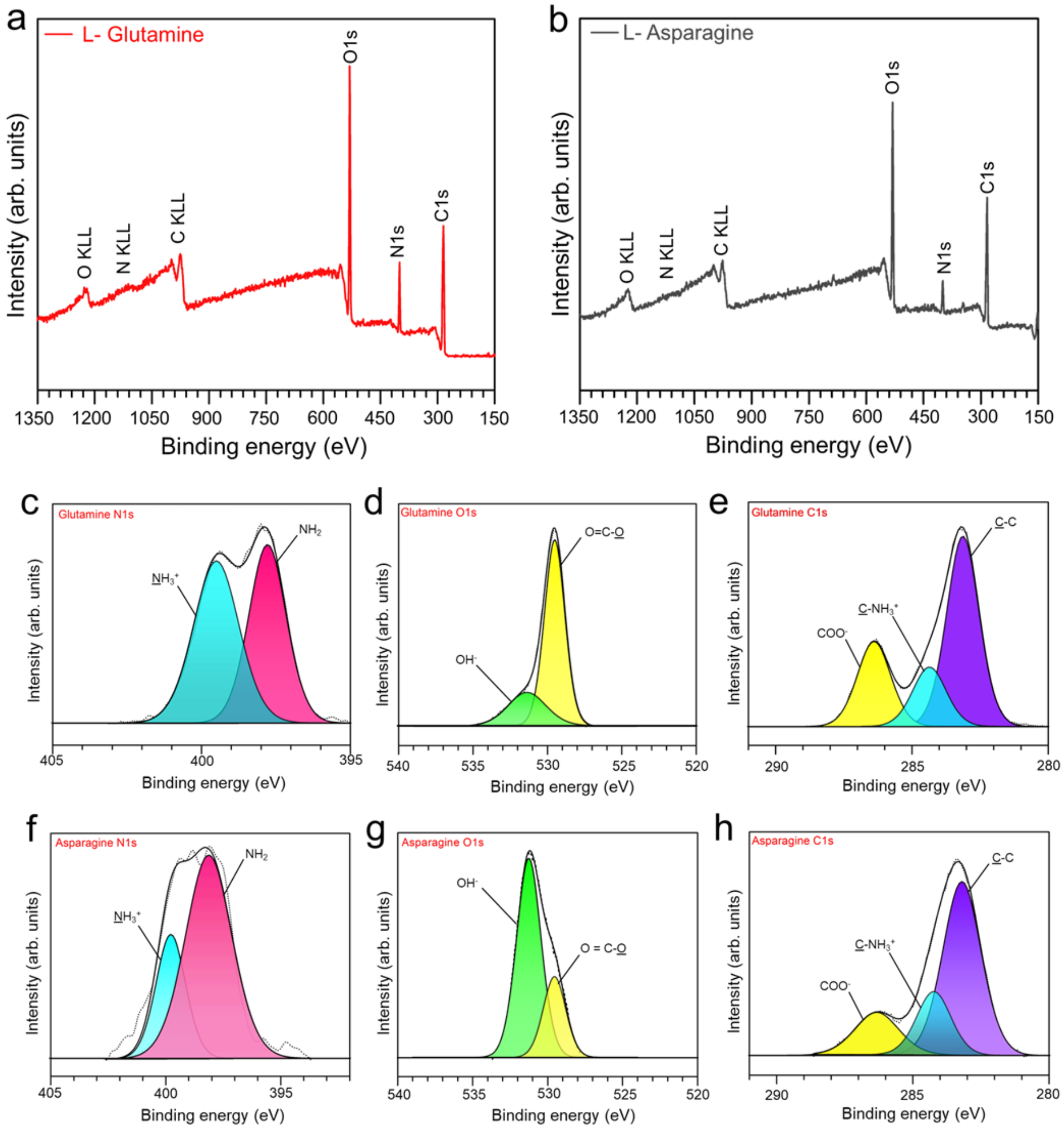

Figure S8. XPS characterization of amino acids with polar uncharged sidechains (amide sidechains). Survey spectra of a) L-glutamine and b) L-asparagine. High-resolution core level photoemission spectrum c) N1s of L-glutamine, d) O1s of L-glutamine, e) C1s of L-glutamine, f) N1s of L-asparagine, g) O1s of L-asparagine, and h) C1s of L-asparagine. Herein, black dots represent experimental data, whereas by summing the Gaussian–Lorentzian fits related to respective components, the final fit was obtained and represented by the continuous bold black line (various moieties are represented in different colors under the spectra).

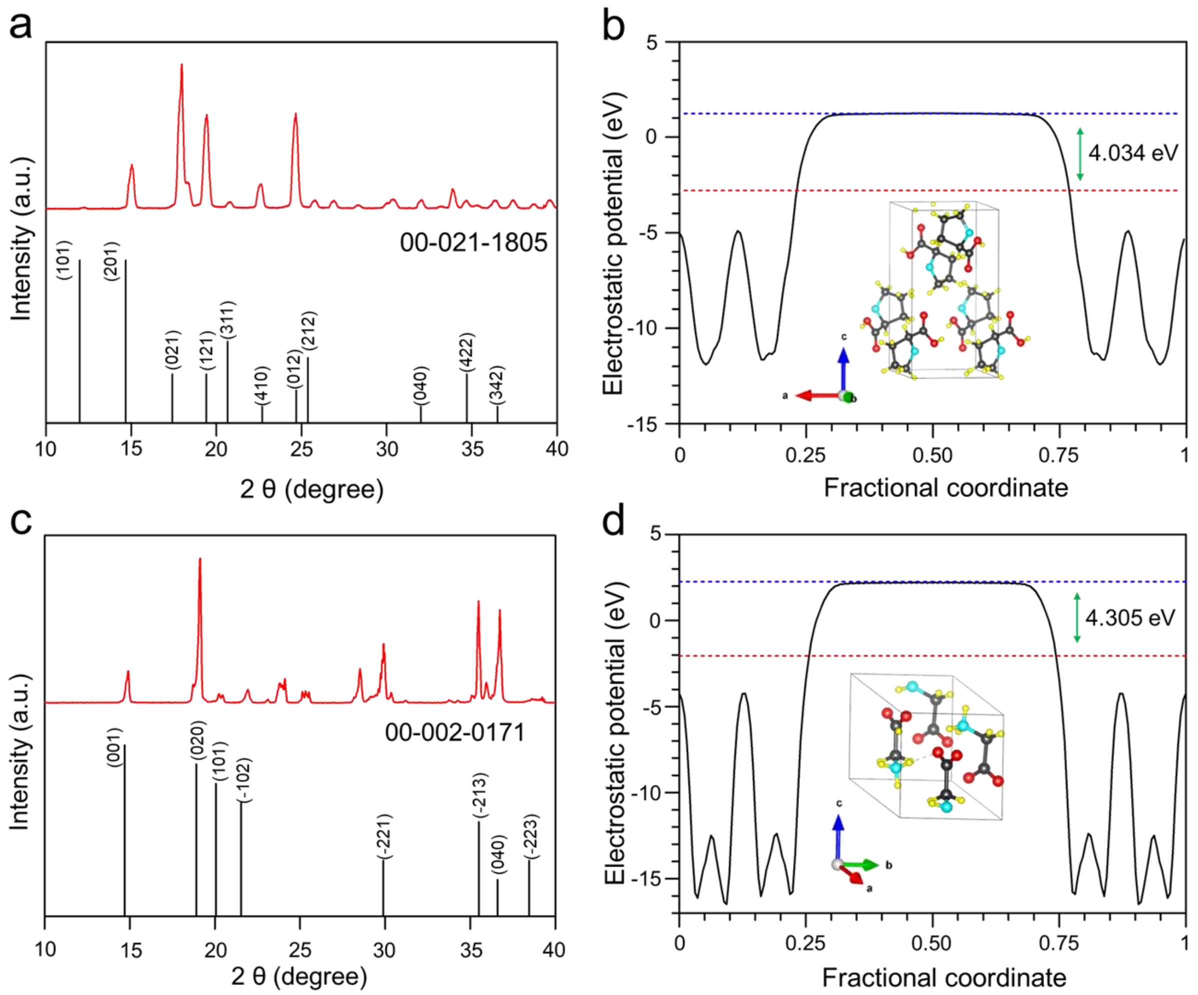

Figure S9. Characterization and work function estimation by DFT calculations. XRD patterns of a) L-proline and c) glycine. Simulated work functions and the unit-cell structures of b) L-proline (0 2 1) and d) Glycine (0 0 1).

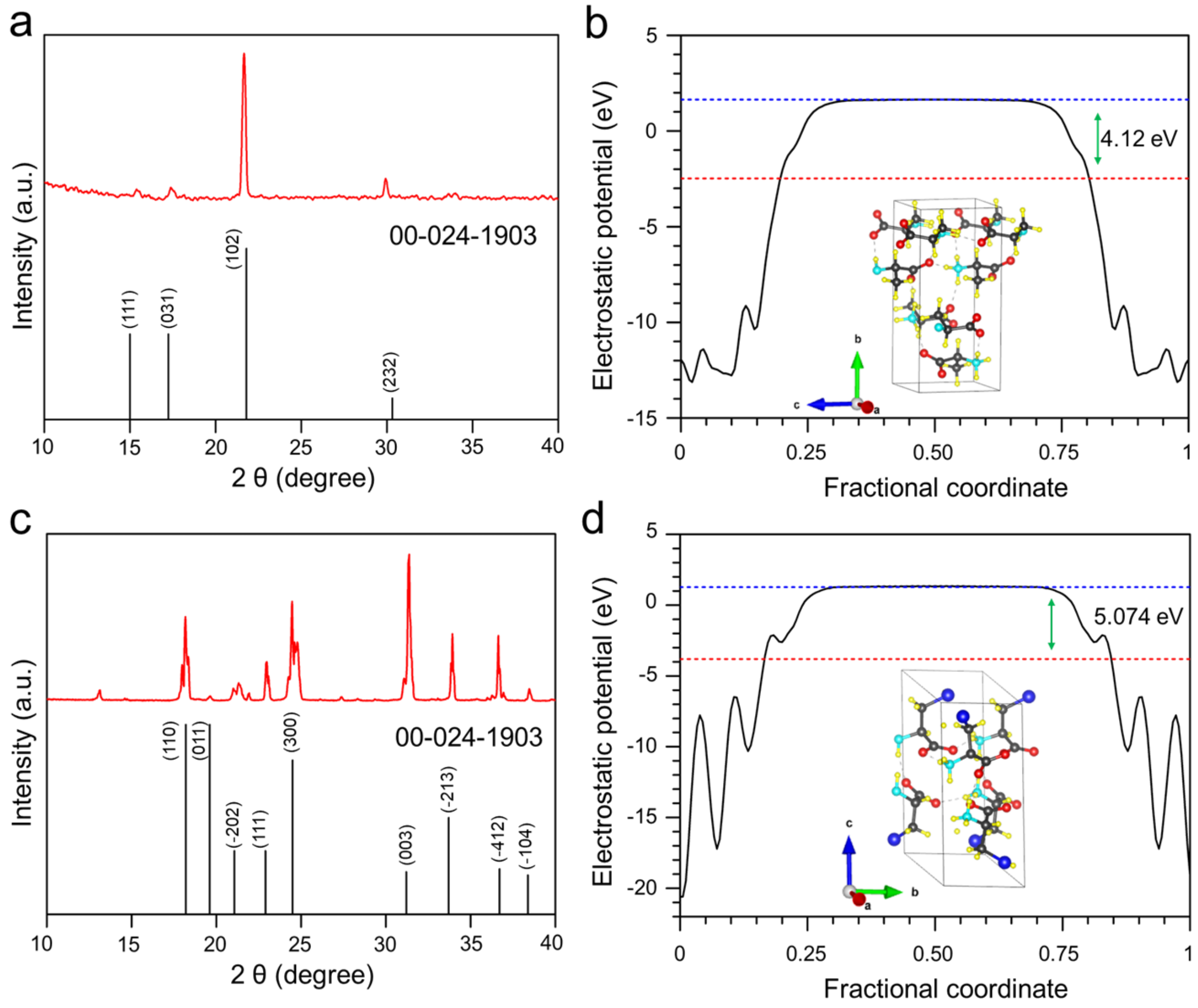

Figure S10. Characterization and work function estimation by DFT calculations. XRD patterns of a) L-alanine and c) L-cysteine. Simulated work functions and unit-cell structures of b) L-alanine (1 1 1) and d) Cysteine (0 0 3).

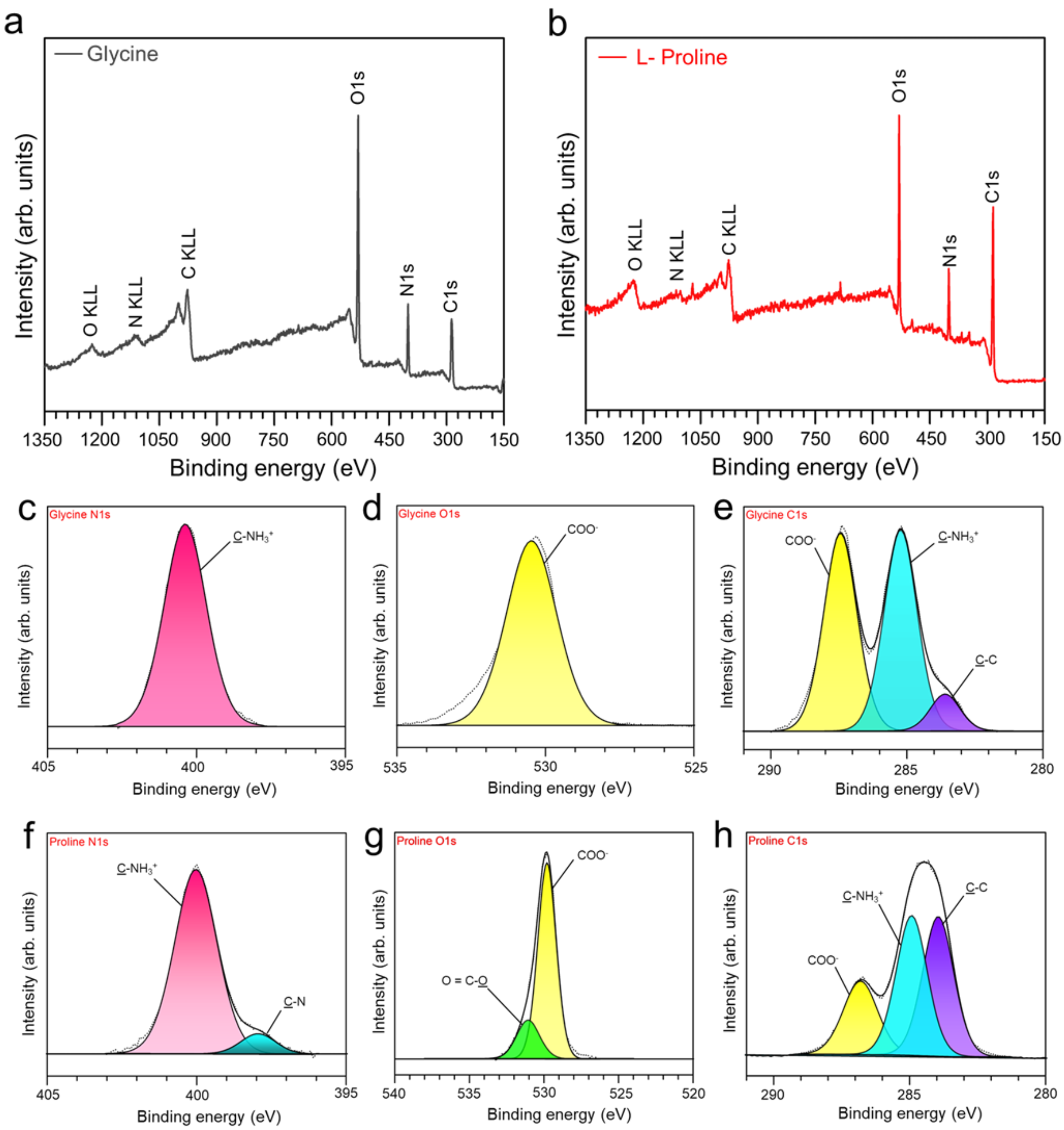

Figure S11. XPS characterization of amino acids with special and hydrophobic sidechains. Survey spectra of a) Glycine and b) L-proline. High-resolution core level photoemission spectrum c) N1s of glycine, d) O1s of glycine, e) C1s of glycine, f) N1s of L-proline, g) O1s of L-proline, and h) C1s of L-proline. Herein, black dots represent experimental data, whereas by summing the Gaussian–Lorentzian fits related to respective components, the final fit was obtained and represented by the continuous bold black line (various moieties are represented in different colors under the spectra).

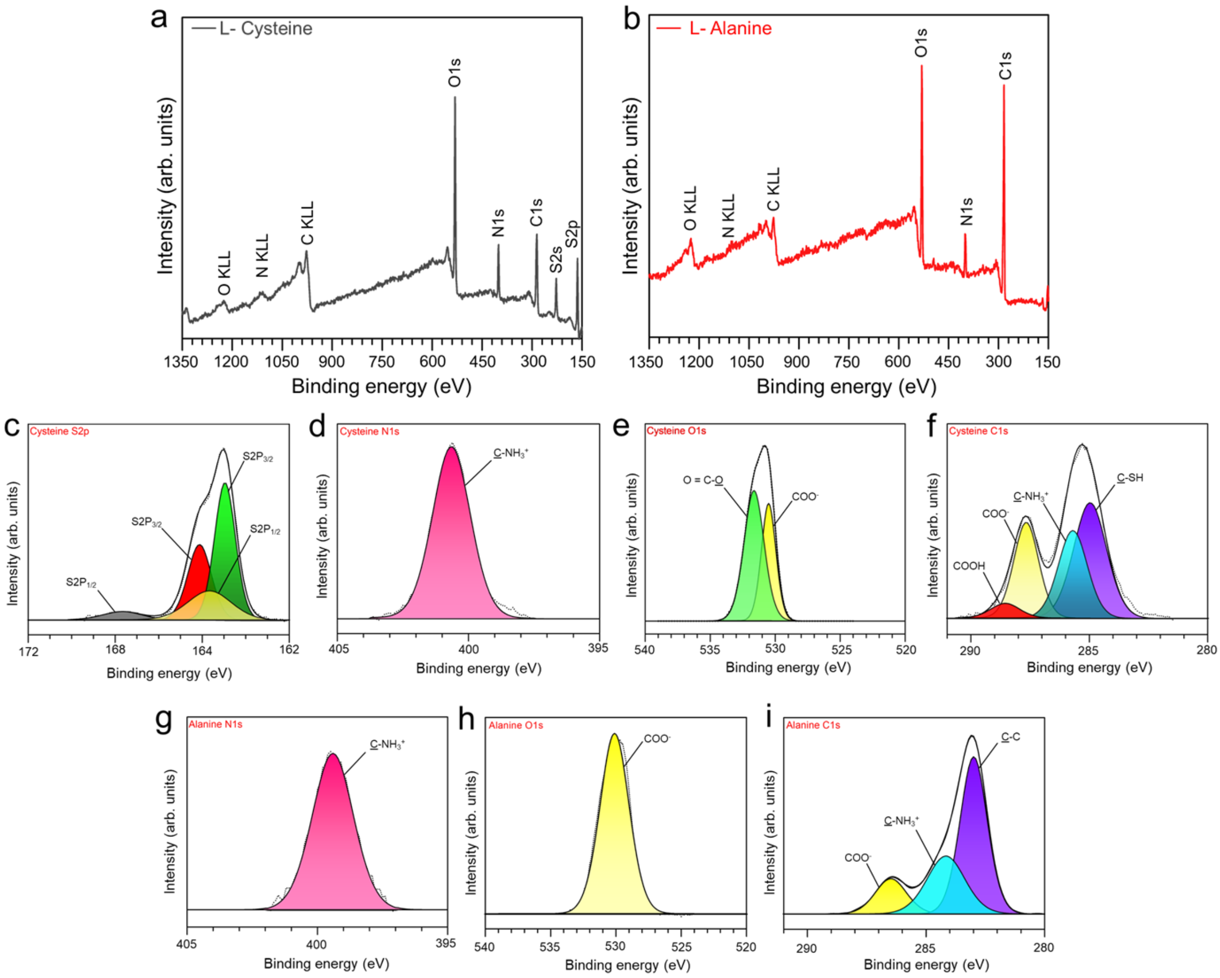

Figure S12. XPS characterization of amino acids with special and hydrophobic sidechains. Survey spectra of a) L-cysteine and b) L-alanine. High-resolution core level photoemission spectra c) S2p of L-cysteine, d) N1s of L-cysteine, e) O1s of L-cysteine, f) C1s of L-cysteine, g**)** N1s of L-alanine, and h) O1s of L-alanine, i) C1s of L-alanine. Herein, black dots represent experimental data, whereas by summing the Gaussian–Lorentzian fits related to respective components, the final fit was obtained and represented by the continuous bold black line (various moieties are represented in different colors under the spectra).

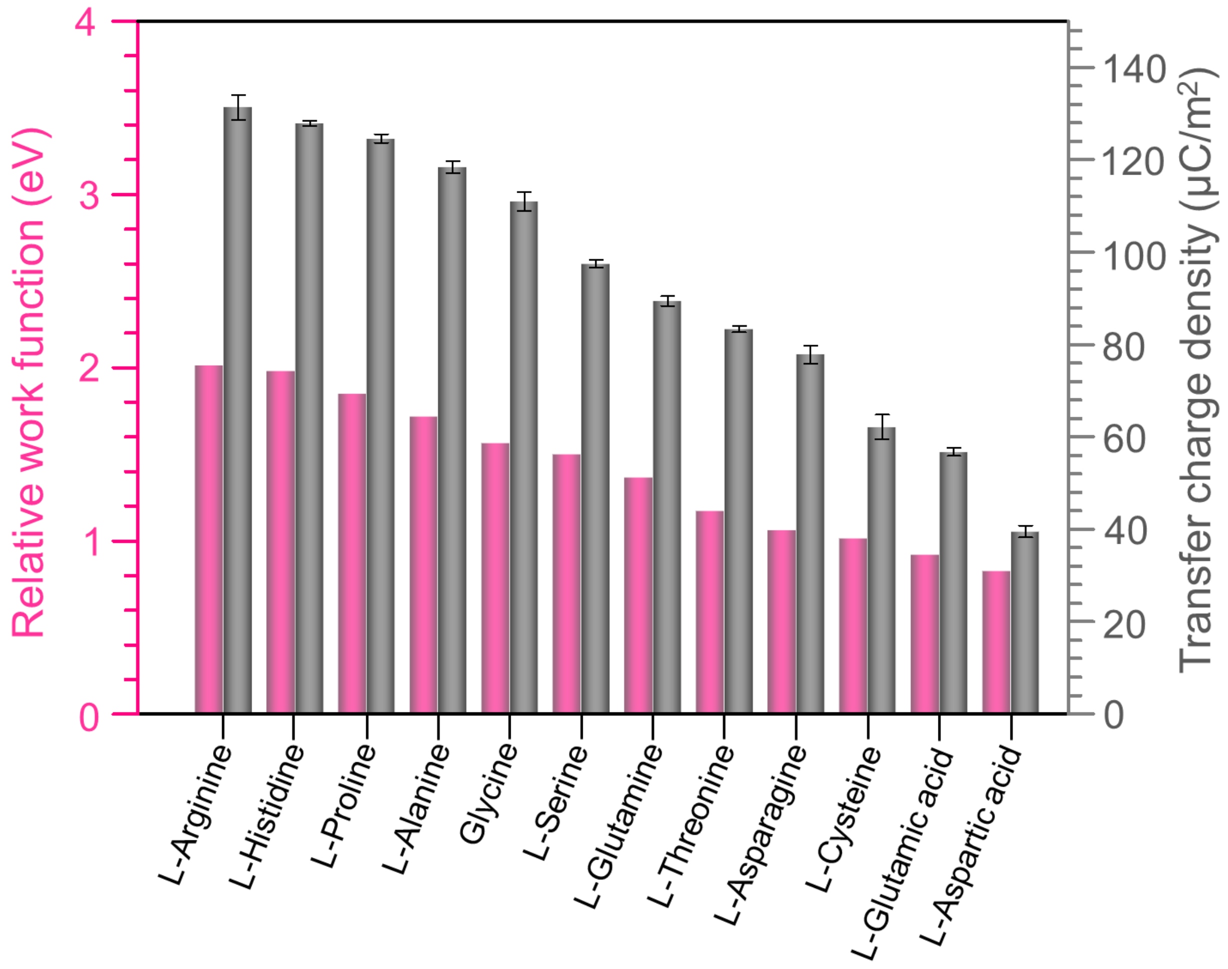

Figure S13. Relationship between the transfer charge density during contact electrification and the relative work functions of the amino acids with PTFE.

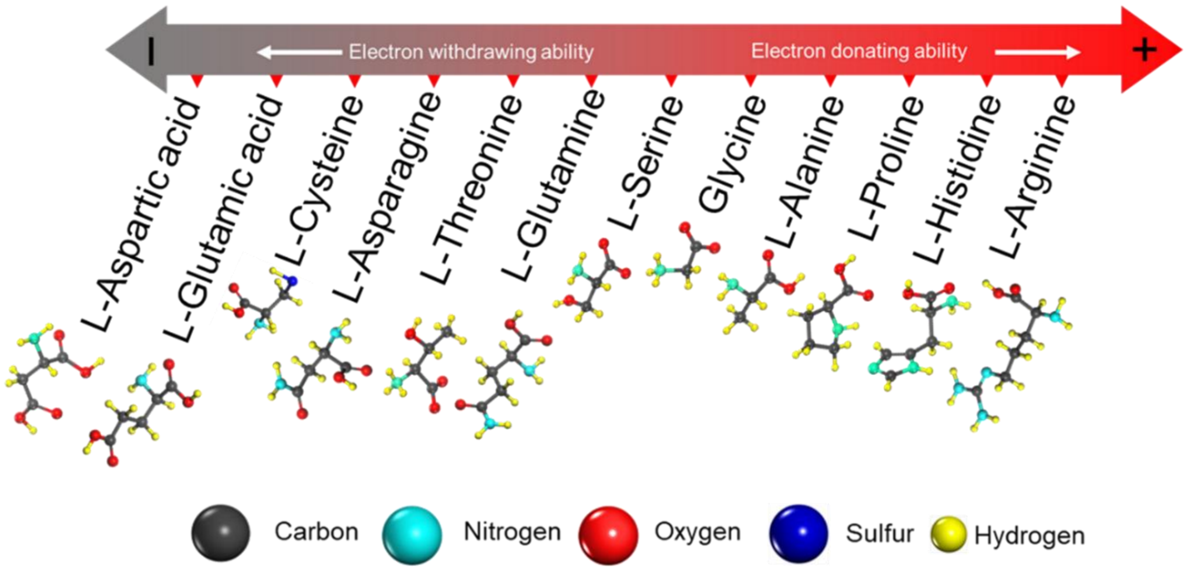

Figure S14. The triboelectric series of amino acids.

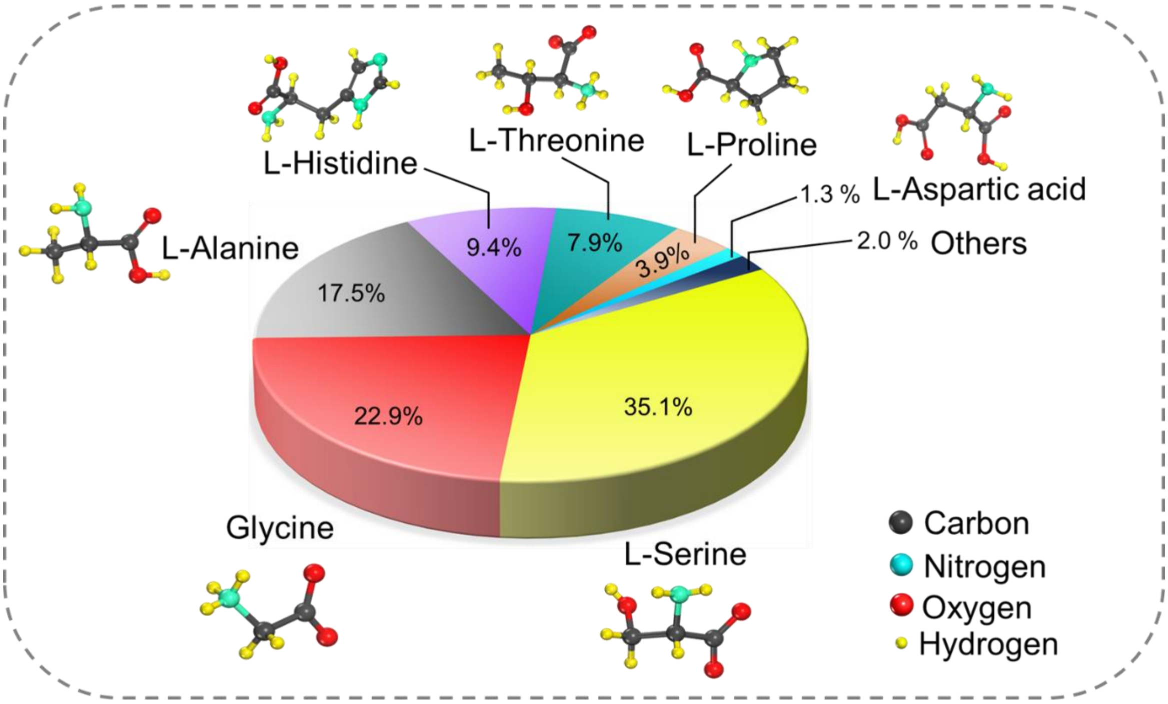

Figure S15. Pie chart representation of the constituent amino acids of the human stratum corneum. [1,3]

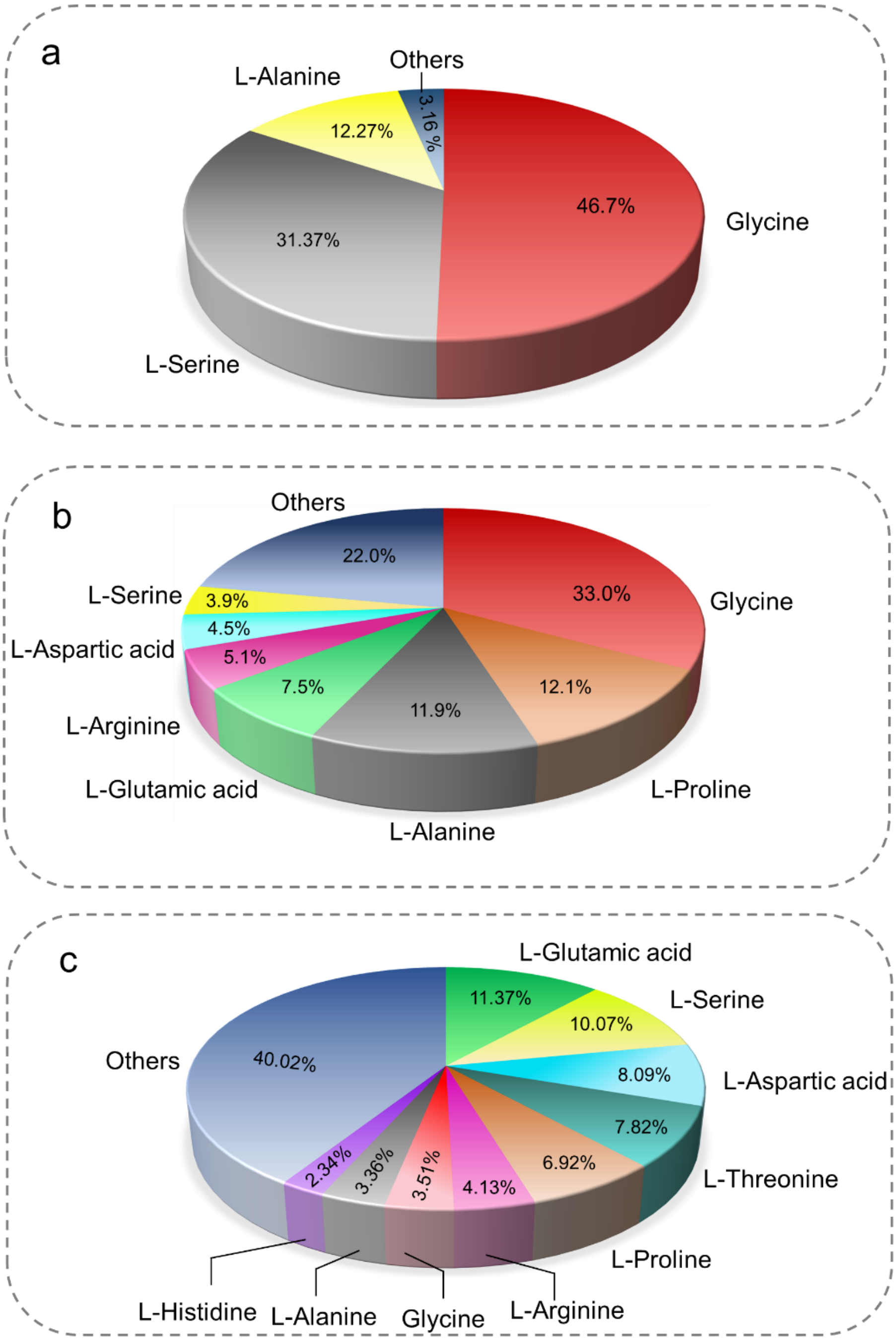

Figure S16. Pie chart representation of the constituent amino acids of proteins. a) Silk fibroin [4-8], b) calf skin collagen [9-11], and c) gamma globulin. [12-14]

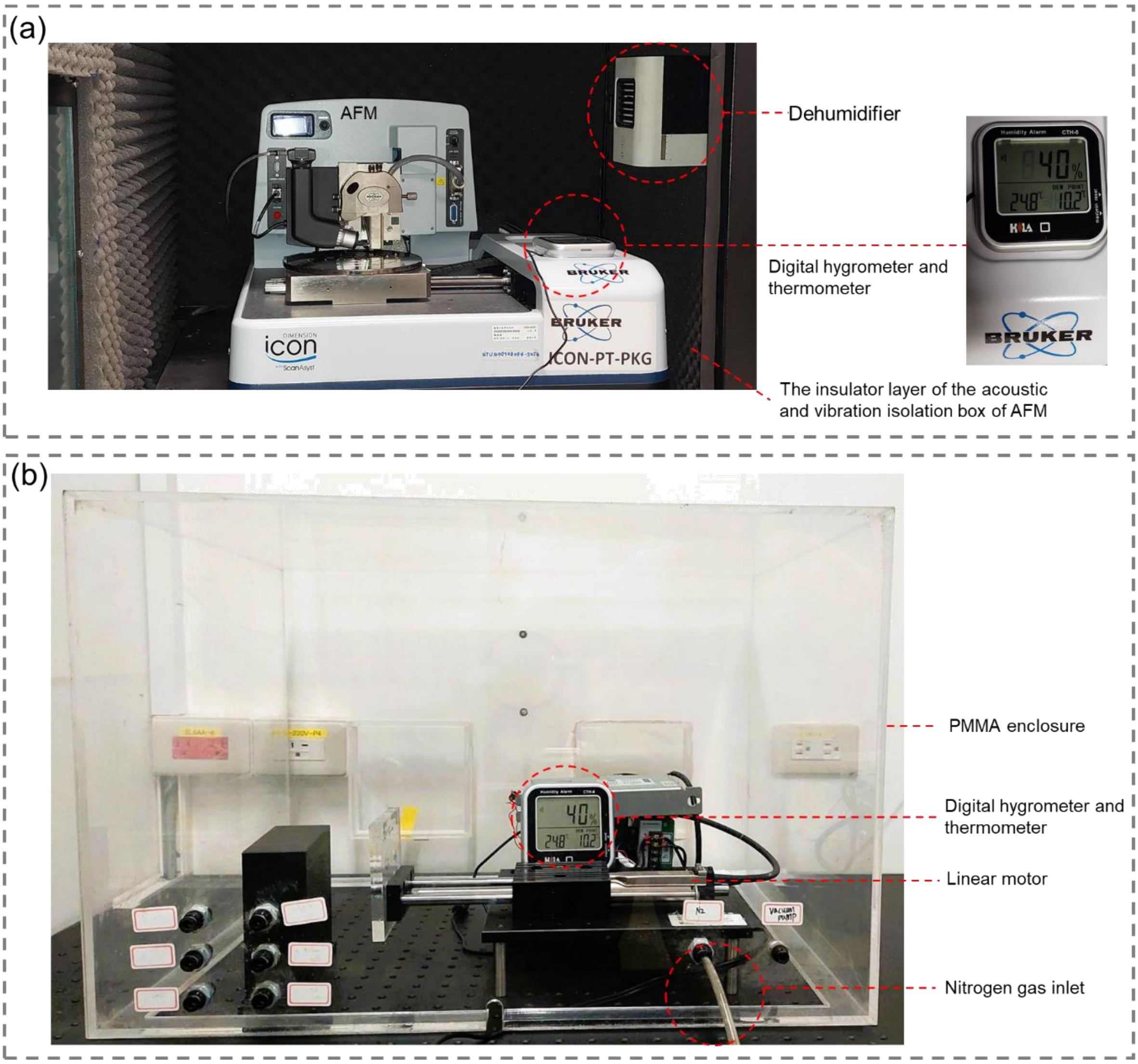

Figure S17. Experimental setup. a) digital image of atomic force microscopy (AFM) system with the dehumidifying system, b) digital image of the electrical output measurement system.

Reference

[1] J.-P. Sylvestre, C.C. Bouissou, R.H. Guy and M.B. Delgado-Charro, *Br. J. Dermatol.* **2010** 163, 458–465.
[2] I. Horii, Y. Nakayama, M. Obata, H. Tagami, *Br. J. Dermatol.* **1989**,121, 587-59.
[3] J.-H. Kim, B. Ahn, S.-G. Choi, S. In, A. R. Goh, S.-G. Park, C.-K. Lee, N.-G. Kang, *PLOS ONE.* **2019**, 14, e0215244.
[4] S.-H. Park, T. Song, T. S. Bae, G. Khang, B. H. Choi, S. R. Park, B.-H. Min, *Int. J. Precis. Eng. Manuf.* **2012**, 13, 2059.
[5] P. Kittiphattanabawon, S. Nalinanon, S. Benjakul, H. Kishimura, *J. Chem.* **2015**, 2015, 1.
[6] F. Yu, C. Zong, S. Jin, J. Zheng, N. Chen, J. Huang, Y. Chen, F. Huang, Z. Yang, Y. Tang, G. Ding, *Mar. Drugs* **2018**, 16, 29.
[7] S. Faralizadeh, E. Z. Rahimabadi, S. H. Bahrami, S. Hasannia, *Sustain. Chem. Pharm.* **2021**,22, 100454.
[8] M. Blanco, N. Sanz, R. I. Pérez-Martín, C. G. Sotelo, *Protein Expr. Purif.* **2023**, 212, 106356.

[9] A. R. Murphy, D. L. Kaplan, *J. Mater. Chem.* **2009**, 19, 6443.
[10] A. Vasconcelos, G. Freddi, A. Cavaco-Paulo, *Biomacromolecules* **2009**, 10, 1019.
[11] M. Li, M. Ogiso, N. Minoura, *Biomaterials* **2003**,24, 357–365.
[12] Y. Z. Lee, J. S. Sim, S. Al-Mashikhi, S. Nakai, *J. Agric. Food Chem.* **1988**,36,5.
[13] A. L. Shaw, D. W. Mathews, J. E. Hinkle, B. W. Petschow, E. M. Weaver, C. J. Detzel, G. L. Klein, T. P. Bradshaw, *Clin. Exp. Gastroenterol.* **2016**, 9, 365.
[14] W. T. Roubal, A. L. Tappel, *Arch. Biochem. Biophys.* **1966**,113(1), 5-8.
